# Health systems and global progress towards malaria elimination, 2000–2016

**DOI:** 10.1186/s12936-020-03208-6

**Published:** 2020-04-08

**Authors:** Maitreyi Sahu, Fabrizio Tediosi, Abdisalan M. Noor, John J. Aponte, Günther Fink

**Affiliations:** 1 Swiss Tropical and Public Health Institute and University of Basel, Basel, Switzerland; 2 University of Washington, Seattle, USA; 3 Global Malaria Programme, World Health Organization, Geneva, Switzerland

**Keywords:** Malaria, Health systems, Elimination

## Abstract

**Background:**

As more countries progress towards malaria elimination, a better understanding of the most critical health system features for enabling and supporting malaria control and elimination is needed.

**Methods:**

All available health systems data relevant for malaria control were collated from 23 online data repositories. Principal component analysis was used to create domain specific health system performance measures. Multiple regression model selection approaches were used to identify key health systems predictors of progress in malaria control in the 2000–2016 period among 105 countries. Additional analysis was performed within malaria burden groups.

**Results:**

There was large heterogeneity in progress in malaria control in the 2000–2016 period. In univariate analysis, several health systems factors displayed a strong positive correlation with reductions in malaria burden between 2000 and 2016. In multivariable models, delivery of routine services and hospital capacity were strongly predictive of reductions in malaria cases, especially in high burden countries. In low-burden countries approaching elimination, primary health center density appeared negatively associated with progress while hospital capacity was positively correlated with eliminating malaria.

**Conclusions:**

The findings presented in this manuscript suggest that strengthening health systems can be an effective strategy for reducing malaria cases, especially in countries with high malaria burden. Potential returns appear particularly high in the area of service delivery.

## Background

To achieve a world free of malaria, health systems in malaria-endemic countries face a series of challenges: they need to identify populations at risk, cover at-risk populations with effective preventive interventions, accurately diagnose and report cases, and treat malaria patients with timely and high-quality care. Constraints to delivery of essential services not only cause inefficiencies, but can also hinder progress in malaria control [1–3]. Deficiencies in national health and surveillance systems likely contributed to the failure of the Global Malaria Eradication Programme 50 years ago [4]. As a result of lessons learned, ongoing elimination and eradication initiatives for other diseases have made a more purposeful effort to utilize and build upon existing service delivery mechanisms [5, 6], and the World Health Organization (WHO) Global Technical Strategy for Malaria now recommends that national strategic plans consider health systems readiness to expand malaria programmes [7]. The recent renewed enthusiasm for malaria eradication [8–10] has rekindled the dialogue and interest in whether present-day health systems are prepared for this bold endeavour [11].

Health system strength generally has not been a primary consideration in decisions to launch disease eradication programmes, which have traditionally focused on biological and technical factors, as well as costs and political will [12, 13]. Research and discourse on this topic has primarily focused on understanding the effects that vertical programmes have had on health systems [14, 15] and on outlining approaches to ensure that eradication activities confer maximum benefits to health systems while maintaining overall programmatic goals [13, 16]. In 2010, several studies were published on the technical and operational feasibility of malaria elimination. Most of these studies focused on strategies and needs for interrupting transmission, and on assessing countries’ ability to create effective national malaria programmes at scale [17–21]. The recent Lancet Commission on Malaria Eradication proposes an ambitious target of eradicating malaria by 2050. This report recognizes that a successful programme will need to strengthen health systems capacity by increasing human resources and developing management competency at the provincial level, but highlights that historically malaria elimination has been achieved in many countries well before health systems have provided universal coverage [9].

To date, however, there has been limited quantitative analysis of the role of health systems in prior malaria control efforts, and for elimination programmes in particular. Measuring health systems performance is complex given the multi-dimensional nature of these systems and the variable importance of these dimensions in different contexts [22–24]. Over the last 15 years more data on health systems have become available at the global level, expanding the ability to characterize and compare health systems across countries. The main objective of this study was to utilize these datasets to empirically assess which aspects of health systems have been most predictive of progress towards malaria elimination from 2000 to 2016.

## Methods

The conceptual approach for this study focused on identifying characteristics of health systems most predictive of progress in malaria control conditional on contextual factors, and is presented in Additional file 1: Analytical Framework. Contextual factors included the level of development of the country, initial malaria burden and broad resource availability to control for level of investments in the health sector. This framework was developed through review and feedback during meetings of the Strategic Advisory Group on malaria eradication in 2018–2019.

### Data sources

All available country-level information on health systems in the 2000–2016 period was extracted and combined in a new health system database. A total of 23 online data repositories related to malaria control and health systems were reviewed for relevant variables, including the World Development and Governance Indicators, the WHO Global Health Observatory, the World Bank Health Nutrition and Population database and country-level data provided through the Measure DHS programme [25–32]. Additional file 2 gives a full list of all data sources for which variables were reviewed for inclusion in the analysis.

### Predictor variable identification and classification

A total of 83 health systems variables were grouped broadly into six categories following the building blocks of the WHO health system framework: health system financing, health service delivery, access to medicines, health system workforce and capacity, governance, and information systems [22]. Additionally, 35 macro-economic, demographic and geographic indicators relevant for health systems and malaria control were reviewed for inclusion in the analysis as control variables. Variables covering less than 50% of the core sample of 105 malaria-endemic countries were excluded from the analysis, and variables were also qualitatively reviewed for inclusion based on relevance to the analytical framework, as well as redundancy with other variables. Additional file 3 provides an overview of domain coverage for all variables reviewed for inclusion in the analysis, and Additional file 4 gives full definitions for all variables included in the analysis. Period averages for health systems variables were computed across the 2000–2016 period.

### Outcome variables

In general, the outcome used for the regression analysis was the relative reduction in malaria cases per 1000 population (for *Plasmodium falciparum* and *Plasmodium vivax* combined) over the 2000–2016 period, obtained from the World Malaria Report [33]. Malaria incidence, rather than mortality, was used because it better aligned with the primary question of interest on progress towards elimination. In addition, many of the malaria mortality statistics published in the World Malaria Report are modelled by applying a static case fatality of 0.256%, thus containing limited information beyond the underlying case data [33]. In the absence of reliable mortality data, parasite prevalence reductions could have been a viable alternative in principle. However, this was not possible in practice because parasite prevalence estimates are modelled using several health systems covariates as inputs, including health expenditure, antenatal care coverage, immunization coverage, and treatment-seeking behaviour [34, 35]. The primary outcome considered in this analysis was thus the reduction in the number of malaria cases per 1000 people over the 2000–2016 period. Among low-burden countries (< 1 case per 1000 in 2000), a binary proxy for elimination of whether the country reached 0 cases by 2016 was also analysed, using unadjusted surveillance data [33].

### Preliminary (single variable) analysis

In a first step, all health systems variables (period averages) extracted for inclusion in the analysis were correlated individually in unadjusted and adjusted regressions with absolute reductions in malaria cases between 2000 and 2016 as the outcome. Adjusted regressions controlled for initial level of development as measured by the Human Development Index (HDI), an indicator which includes income, education and life expectancy, and level of malaria burden (low, medium or high) in 2000 [32]. Regressions adjusted for HDI were also performed separately within the three malaria-burden categories.

### Data consolidation

Given that several variables were identified as proxies for the same health system aspects or concepts, health system variables were consolidated into core domains for the main analysis. These domains were broadly defined using the WHO building block framework [22] except that health financing variables were considered as a contextual factor rather than a proxy of health system functioning because they generally capture broad resource availability which may affect all domains of health systems (Additional file 1). Using the final health systems dataset, any remaining missing data at the country level were imputed using multiple imputation chained equations. Additional file 5 shows descriptive statistics for all 35 health systems variables in the original and imputed datasets. Principal component analysis was then used to consolidate the available data within each domain into core components of health systems. In cases where the first principal component accounted for the majority of total domain variation observed (> 50%), only the first component was retained. If the first component explained less than 50%, additional sub-components were constructed until more than half of variation in each component was accounted for. Composite ‘health systems scores’ were generated by grouping countries into deciles for each component and summing deciles across domains.

### Main analysis

First, health systems scores (summed scores and rankings of countries by their scores) were plotted against progress in malaria control. In addition, the health systems features of countries performing at the top and bottom of their respective burden categories were visually compared.

To identify the most predictive health system components for successful malaria control conditional on initial malaria burden, initial HDI and average total health expenditure as percentage of GDP during 2000–2016, three empirical approaches were used: (1) standard regression models including all 7 health systems components in the regression independent of the strength of their association with progress in malaria control; (2) backward stepwise selection (chosen among iterative search methods because it performed the best in terms of residual error and parsimony, as described further in Additional file 6); and, (3) a systematic combinatory approach which searched through every possible combination of the 7 components (127 regressions in all) to minimize residual error. All models were estimated using linear regression with heterogeneity-robust standard errors [36, 37].

Given that the relative importance of specific health system factors likely varies across different stages of malaria control, separate models for countries with high initial incidence (≥ 300 cases per 1000 in 2000), moderate initial incidence (1–299 cases per 1000 in 2000) and low initial incidence (< 1 case per 1000 in 2000) were fitted.

## Results

**Fig. 1:**
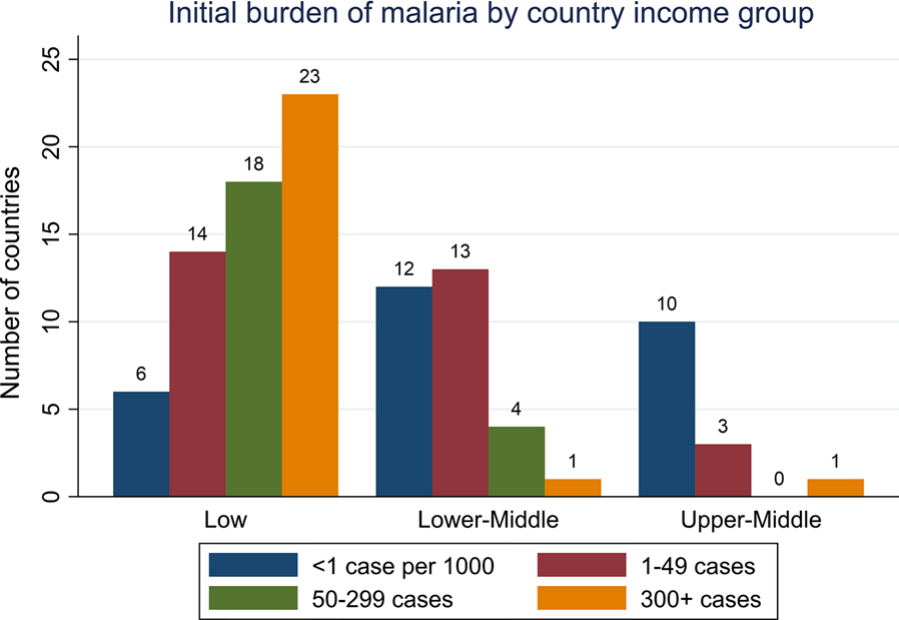
Initial burden of malaria by country income group. This figure shows the initial distribution of the 105 sample countries with respect to World Bank Income Category and initial malaria burden

As of 2000, malaria existed primarily in low and lower-middle income countries. As Fig. 1 illustrates, 61 out of the 105 countries with at least one malaria case after 2000^1^ were classified as low-income, and 30 of these countries were classified as lower-middle income. Table 1 gives an overview of baseline and average malaria case burden and development level in the 2000–2016 period. Mean gross domestic product (GDP) per capita in this group increased from $4404 to $8454 during this period,^2^ and countries on average reduced their case burden by 60% (Table 1).

**Table 1:** Descriptive statistics for countries with at least one case of malaria after 2000 (N = 105)

	Baseline (2000)		Endline (2016)	
	Mean	95% CI	Mean	95% CI
Malaria cases per 1000	131.6	(97.9, 165.3)	78.8	(55.1, 102.5)
GDP_pc_, PPP^a^	$4402.5	($3290.6, $5514.5)	$8453.5	($6724.0, $10,183.0)
HDI^b^	51.8	(49.0, 54.6)	60.0^c^	(57.5, 62.5)

^a^GDP per capita based on Purchasing Power Parity (in current international $)

^b^Human Development Index (HDI) is based on GDP, life expectancy and education

^c^HDI endline data are from 2015, the last year available in this dataset

### Individual health systems variables and progress in malaria control

Out of 51 unique health system and financing variables identified, 26 variables showed statistically significant associations (p < 0.05) with reduction in malaria cases from 2000 to 2016 in univariate models. However, when models were adjusted for initial malaria burden and HDI in 2000, only five variables displayed statistically significant associations with progress in malaria control: measles immunization coverage, tuberculosis treatment success rate, coverage of insecticide-treated nets (ITNs) among the high-risk population, children with fevers seeking care in the public sector, and malaria surveillance reporting completeness. Additional file 7 summarizes these results. When stratifying the adjusted models by initial burden of malaria, the strongest associations were in the highest burden group (> 300 cases per 1000) across four domains: overall health and malaria-specific financing, health service delivery, access to medicines, and health workforce and capacity (Additional file 8). Among high-burden countries only, the following health spending variables displayed positive correlations with reductions in malaria burden: percent of total health expenditure which is from foreign aid, external malaria financing per capita, and external malaria financing allocated specifically for ITNs, treatment, diagnosis, and health system strengthening.

### Principal component analysis

Table 2 shows the results of principal component analyses within each domain of health systems, and Additional file 5 gives the full details and descriptive statistics for each of the variables included for each domain. A total of 7 principal health system components were identified, ranging from health service delivery to health information systems.

**Table 2:** Health systems components identified in principal component analysis (PCA)

No.	Name of component	No. of variables included	% of domain variance explained
1	Health service delivery, routine services	6	60.1
2	Access to medicines	2	68.3
3	Health workforce	3	74.6
4	Health system capacity: basic health centers	2	91.6
5	Health system capacity: hospital capacity	2	56.1
6	Governance	8	64.8
7	Health information systems	2	74.2

### Health systems scores

**Fig. 2:**
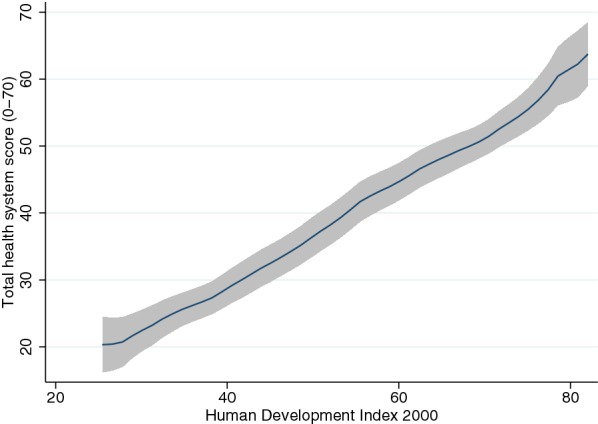
Health systems score and initial level of development in 2000. This figure shows kernel-weighted local polynomial smoothed relationship between the Human Development Index and total health systems scores

Total health system scores (the sum of the decile ranks in the 7 domains) ranged from 10 in the country with the weakest health system (Chad) to 69 in the country with the strongest health system (South Korea). This overall score displayed a strong (and mostly linear) relationship with countries’ HDI in 2000 (Fig. 2).

**Fig. 3:**
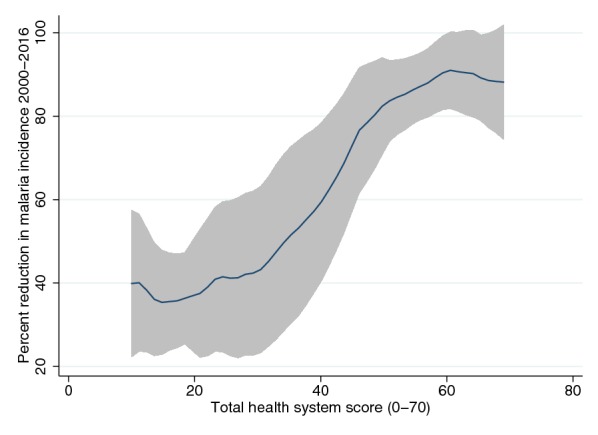
Health systems score and percent reduction in malaria incidence, 2000–2016. This figure shows kernel-weighted local polynomial smoothed relationship between reduction in malaria cases, 2000–2016 and total health systems scores

Health systems scores were also strongly correlated with progress in malaria control between a health systems score of approximately 30 and 60, though the relationship was less strong towards the ends of the scale (Fig. 3).

**Fig. 4:**
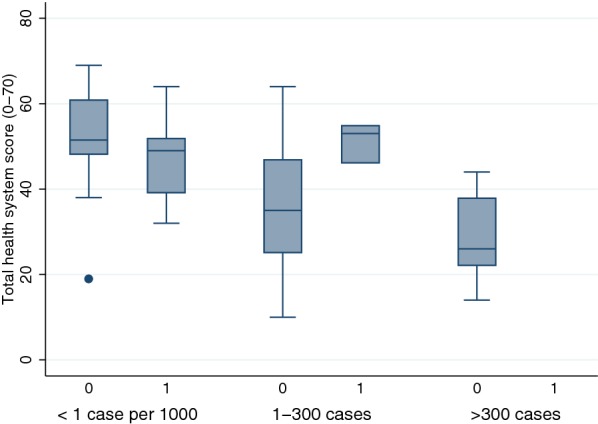
Health systems score for countries which eliminated malaria versus those that did not, by burden category (‘Yes’ = reached 0 cases, ‘No’ = did not reach 0 cases). This figure shows distribution of health systems scores by baseline burden and the likelihood of reaching 0 cases by 2016. In the first group (< 1 case per 1000 initially), 14 countries (50% of countries in this group) reached 0 cases; in the second group (1–300 cases), the same was achieved by 3 countries (6%), and in the third group (> 300 cases) no countries reached 0 cases

When comparing the distribution of health systems scores of countries which reached zero cases of malaria *versus* those which did not (Fig. 4), there were no obvious differences for the low-burden category, which had median health systems scores of 51.5 and 49, respectively. In the middle-burden group, differences in health systems scores were substantial: the median health system score among countries reaching zero cases was 53, compared with 35 in the group which did not eliminate malaria. In the high-burden group, no countries reached zero cases by 2016, but countries on average scored more poorly on health systems compared with countries with lower initial burden.

**Fig. 5:**
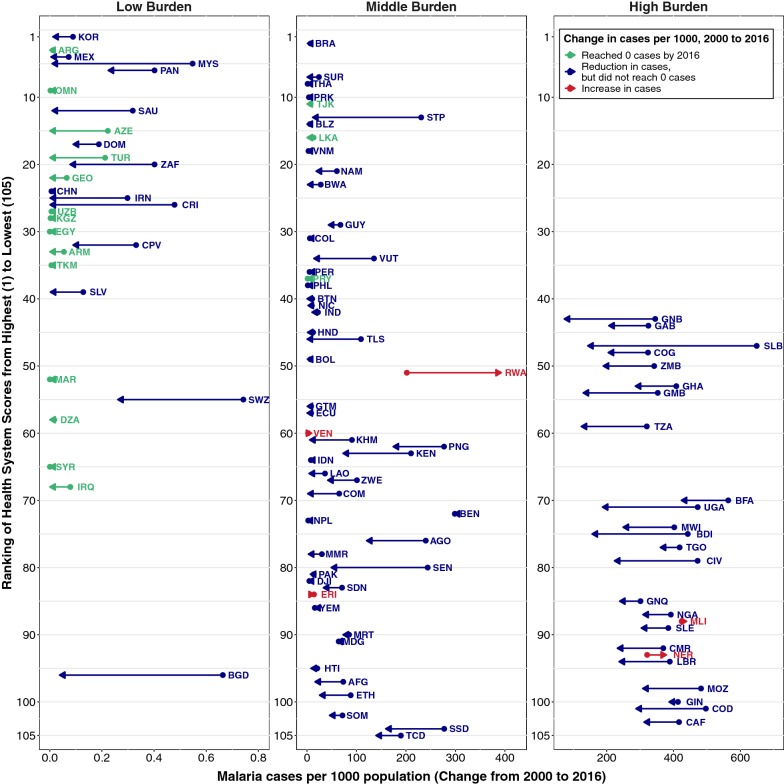
Ranking of health systems score and change in malaria incidence, 2000–2016. Low-burden countries (< 1 case per 1000 in 2000), middle-burden (1–300 cases in 2000), high-burden (> 300 cases per 1000 in 2000). Full country labels and rankings provided in Additional file 9

When plotting change in malaria cases over time according to the ranking of the health system score of the county, large heterogeneity in progress can be observed across burden levels and health system scores (Fig. 5). In general, low-burden countries at baseline appeared to have relatively strong health systems while high-burden countries appeared to have lower ranking health systems. Within the high-burden category, the funnel shape of the arrows seems to indicate that more highly ranked health systems made greater progress in malaria control compared with weaker systems. There were some remarkable departures from the trend: five countries increased in malaria, including Rwanda which increased by 95% despite having a mid-ranked health system (Additional file 9). A literature search to better understand Rwanda’s increase in cases showed a potential gap in usage of effective interventions, e.g., due to pest infestation and perception about their control [38]; fortunately, Rwanda declined in cases from 2016 to 2017, a positive sign that this trend may be reversing [39]. In addition, there were substantial malaria reductions in countries with health systems ranked on the poorest end of the spectrum across all burden categories, including Bangladesh (BGD, 94%), South Sudan (SSD, 43%), and Democratic Republic of Congo (COD, 42%).

### ‘Best and worst performers’ analysis

When comparing health systems features of countries with major reductions in malaria incidence to those of countries with less progress, substantial differences were found.

**Fig. 6:**
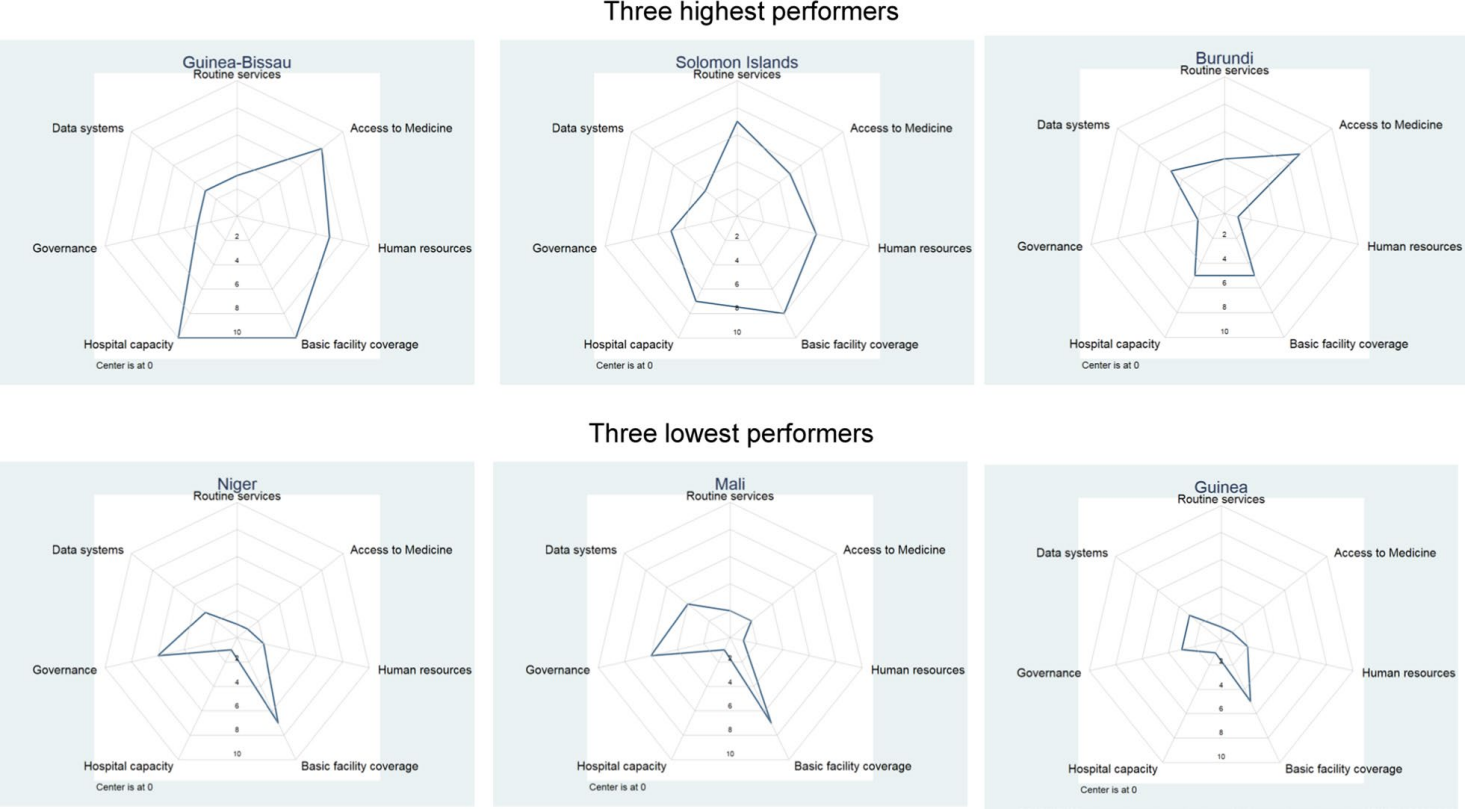
Health systems dimensions for highest and lowest performers for relative change in malaria cases, 2000–2016 among high burden countries (300+ cases per 1000 population)

Figure 6 shows the health systems scores of highest and lowest performers in terms of percentage reduction in the burden of malaria in the high initial burden group. Health systems scores for top performers (Guinea-Bissau, Solomon Islands and Burundi) were overall higher in almost all domains than the health system scores for bottom performers (Niger, Mali and Guinea), though low performers appeared to have reasonably good basic facility coverage.

**Fig. 7:**
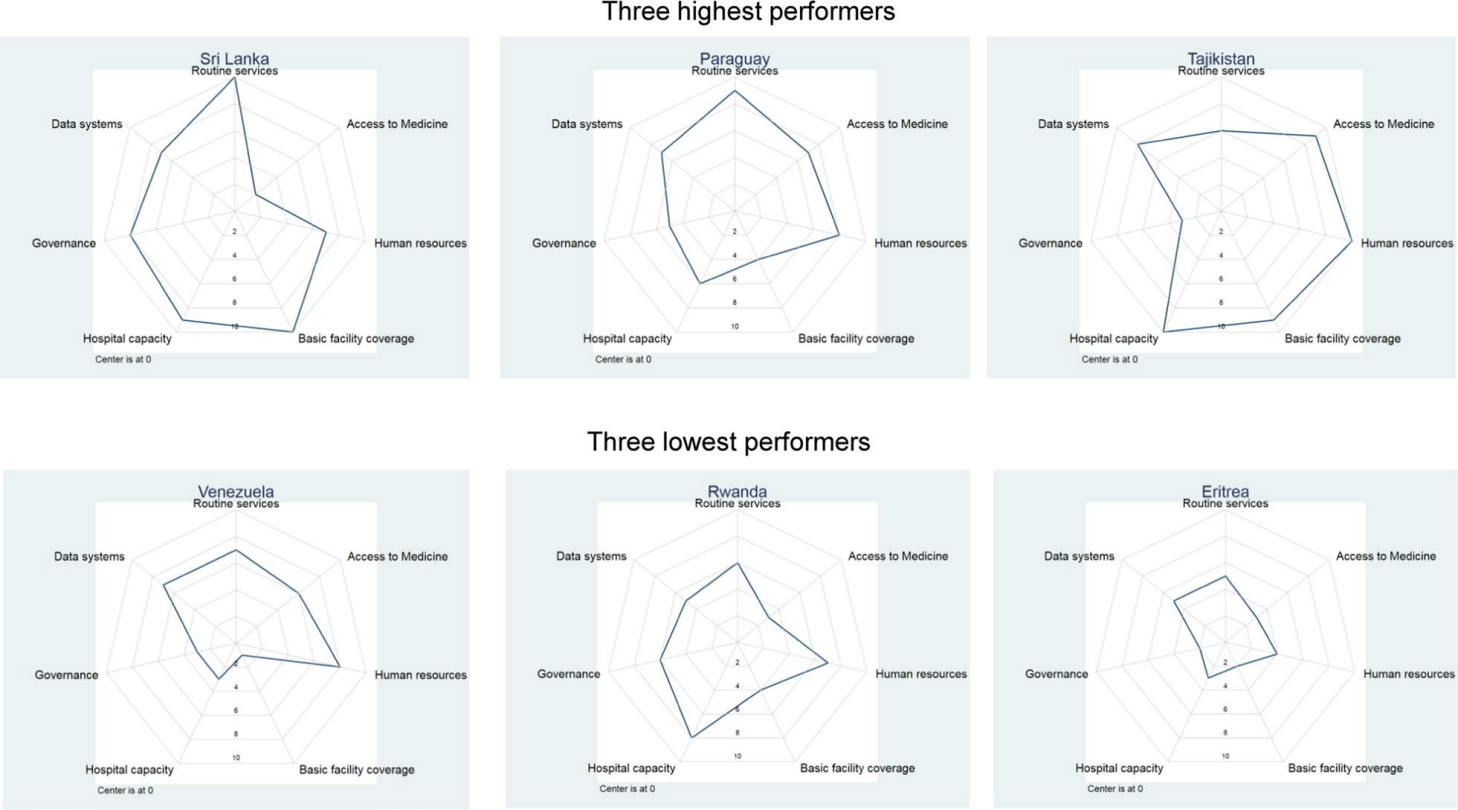
Health systems dimensions for highest and lowest performers for relative change in malaria cases, 2000–2016 among middle-burden countries (1–300 cases per 1000 population)

Figure 7 compares health systems scores of highest and lowest performers among middle-burden countries in 2000. The three most successful countries in this category were Sri Lanka, Paraguay and Tajikistan: all three achieved a 100% reduction in malaria cases, and all had total health systems scores > 45. The low performers had a more complex story. Venezuela scored well on staffing, service delivery and data systems, but had a political and economic situation that deteriorated after 2012 leading to a severe malaria outbreak [40]. Rwanda scored reasonably well in terms of overall health system (score of 39), but regardless experienced a massive increase in malaria burden after 2012 as discussed above. In comparison, Eritrea also experienced an increase in cases but scored poorly on health systems (score of 24).

**Fig. 8:**
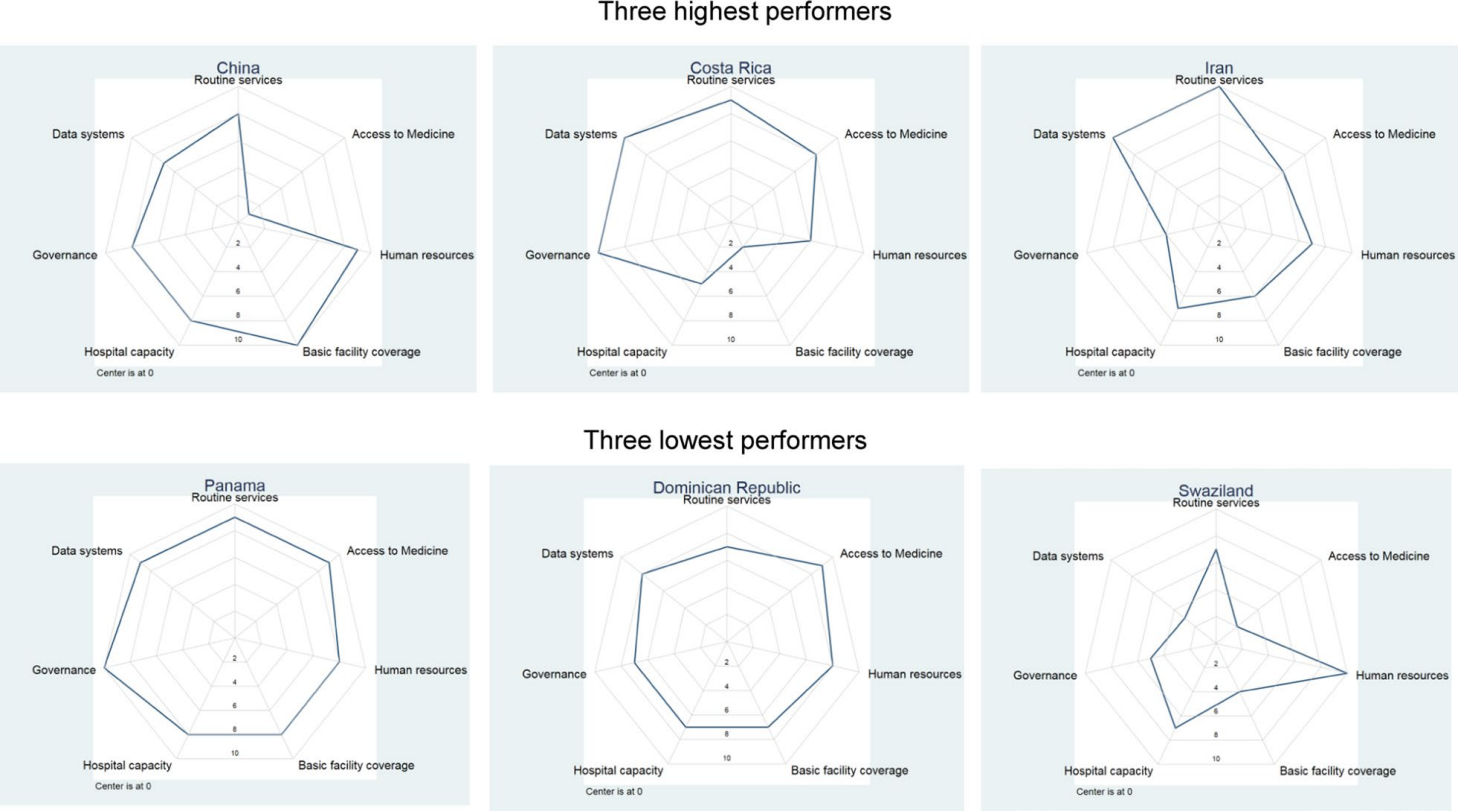
Health systems dimensions for highest and lowest performers for relative change in malaria cases, 2000–2016 among low burden countries (< 1 case per 1000 population), excluding 14 countries which reached 0 cases

Among low-burden countries, little difference was found in the health systems scores between the ‘highest’ and ‘lowest’ performers (Fig. 8); however, every country in this category made large progress towards malaria elimination. The weakest performer in this group was Panama which reduced cases by 44.3%. Additional file 9 provides progress estimates as well as health systems scores for all countries.

### Combined health system regression results

Table 3 shows the main variable selection and regression results using outcomes of percent reduction in cases from 2000 to 2016 (‘malaria control’) and probability of reaching zero cases by 2016 among low-burden countries (‘malaria elimination’). Full model results including p-values are presented in Additional file 10. Column 1 of Table 3 shows results for a basic regression model including all 7 components; columns 2 and 3 show results based on selected variables. The overall predictive power was weak for all malaria control models (R^2^ = 0.17 to 0.20), and only moderately higher when restricted to countries approaching elimination in Columns 4–6 (R^2^ = 0.41 to 0.45). For malaria control (columns 1–3), both backward stepwise and grid search models showed a positive relationship between progress and hospital capacity (p = 0.041 for backwards; p = 0.014 for grid search); however, no health systems features showed a statistically significant relationship with malaria control in the model including all 7 components. For malaria elimination (columns 4–6), greater primary health center density was associated with decreased probability of reaching zero cases (p = 0.027 for all 7 components model; p = 0.014 for backward stepwise and grid search). Hospital capacity was positively correlated with malaria elimination, but this result was only statistically significant in the backwards stepwise and grid search models (p = 0.007). In addition, elimination appeared slightly negatively associated with good governance in all models, though this result was not statistically significant.

**Table 3:** Regression model results (coefficients and R-squared) for malaria case reductions and health system components, 2000–2016

	Health system component	Percent reduction in cases per capita, 2000–2016 (ß)			Reached 0 cases by 2016 (ß) (low-burden countries only)^†^		
	Model selection method	All	Backward stepwise	Grid search	All	Backward stepwise	Grid search
1	Health service delivery, routine services	4.2	5.1	4.9	− 0.05	Not selected	Not selected
2	Access to medicines	4.3	Not selected	Not selected	− 0.13	Not selected	Not selected
3	Health system workforce	0.6	Not selected	Not selected	0.02	Not selected	Not selected
4	Health system capacity: health centres	6.7	Not selected	6.6	− 0.10*	− 0.10*	− 0.10*
5	Health system capacity: hospitals	6.1	9.9*	6.9*	0.18	0.20**	0.20**
6	Governance	1.0	Not selected	Not selected	− 0.05	− 0.08	− 0.08
7	Health information systems	− 0.5	Not selected	Not selected	0.27	Not selected	Not selected

R^2^ for model		0.20	0.17	0.20	0.45	0.41	0.41

All regressions adjust for HDI in 2000 and total health expenditure as percentage of GDP. Percent reduction models (columns I–III) also control for initial malaria burden category. Backward stepwise models exclude variables with p > 0.2 and include variables with p-values < 0.10. Grid search results reflect the model with lowest root mean squared error, among all models including up to 7 components

^†^This analysis was conducted only among countries in the low burden category (< 1 case per 1000 in 2000)

*: Variable is significant at p < 0.05; **: Variable is significant at p < 0.01

In the high burden category, health systems factors predicted progress in malaria control relatively well (R^2^ = 0.71). Table 4 gives the model results stratified by initial malaria burden category. Among high-burden countries, health service delivery and hospital capacity were strongly positively predictive of malaria progress: on average each standard deviation increase in ability to deliver services was associated with an additional 17 percentage point reduction malaria cases (95% CI: 12.2−22.2, p < 0.001). Hospital density and capacity also showed a strong positive correlation with reduction in malaria cases (p < 0.001). Health information systems (including completeness of birth registration and malaria reporting) were negatively associated with malaria progress (p = 0.003), potentially due to some interdependence between completeness of malaria reporting and increased reporting of malaria cases. Among low- and middle-burden countries, health system factors had more limited power in predicting progress in malaria control (R^2^ = 0.26 and 0.16, respectively), and no health systems factors displayed statistically significant associations with progress in malaria control. In low-burden counties, health centre density appeared negatively associated with progress, though this result was not statistically significant at standard cut-offs (p = 0.052).

**Table 4:** Backward stepwise regression model results (coefficients and R-squared) for malaria case reductions and health system components, 2000–2016, stratified by burden category

	Health system component	Percent reduction in cases per capita, 2000–2016 (ß)			Reached 0 cases by 2016 (ß)
	Initial burden category^a^	Low	Middle	High	Low
1	Health service delivery, routine services	Not selected	Not selected	17.2**	Not selected
2	Access to medicines	− 12.4	12.1	Not selected	Not selected
3	Health system workforce	Not selected	Not selected	Not selected	Not selected
4	Health system capacity: health centres	− 2.0	20.0	Not selected	− 0.10*
5	Health system capacity: hospitals	5.6	Not selected	6.6**	0.20**
6	Governance	Not selected	Not selected	− 4.5	− 0.08
7	Health information systems	Not selected	Not selected	− 9.3**	Not selected

R^2^ for backward stepwise model		0.26	0.16	0.70	0.41

All regressions adjust for HDI in 2000 and total health expenditure as percentage of GDP. Backward stepwise models exclude variables with p > 0.2 and include variables with p-values < 0.1

*: Variable is significant at p < 0.05; **: Variable is significant at p < 0.01

^a^Initial burden category is defined as: Low = < 1 case per 1000 in 2000, Middle = 1–300 cases per 1000 in 2000, High = 300+ cases per 1000 in 2000

## Discussion

This study used all currently available quantitative data to identify the health systems factors most relevant for malaria control. While several factors were found to be associated with progress in malaria control, observed associations were highly heterogeneous overall, with no single factor or threshold predicting malaria control progress across all burden groups. In general, countries that were malaria endemic in 2000 and achieved zero malaria cases by 2016 had relatively high health systems scores (Fig. 5). However, when other contextual factors were controlled for, the predictive power of health systems factors was relatively limited (Table 3). The descriptive analysis presented in this paper also makes it clear that rather remarkable progress has been made in several countries with very weak health systems. One of the primary reasons why this may be the case is the largely vertical nature of many national malaria programmes. Prior research shows that the interventions responsible for the greatest malaria reductions during this period have been vector control programmes (foremost distribution of ITNs, as well as indoor residual spraying campaigns) [41], which are often delivered through vertical processes by organizations external to national health systems [42, 43]. These heterogeneous results highlight that context-specific health system strengthening approaches are needed for malaria control and elimination.

Investment in health systems is already considered an important aspect of malaria control programmes [44]. In its eighth round of funding, the Global Fund to Fight AIDS, Tuberculosis and Malaria, which provides 96% of external malaria funding, allocated 37% (US$362 million) of its overall funding towards health systems strengthening, of which US$139 million went towards general health systems support and the remaining went to disease-specific system strengthening [45]. The vast majority (82%) of overall health systems funding went towards three building blocks: service delivery, human resources and medicines [45]. Among low-burden countries approaching elimination, the large majority of external financing in countries goes towards vertical interventions including treatment, diagnosis and vector control, although the portion allocated for health system strengthening is growing [46].

High-burden countries, which generally tend to have weaker health systems, may see the largest benefits from health systems strengthening interventions. Amid recent increases in cases between 2015 and 2017 especially among the highest burden countries [9], revitalization of malaria control approaches are needed in these countries. After restricting the adjusted analysis to high burden countries, health systems displayed strong correlations with progress in malaria control. These findings are inconsistent with a mostly theoretical literature which suggests that research and investments in health systems are likely to have a larger impact in middle-burden countries rather than in low- or high-burden countries [47]. Specifically, among high-burden countries service delivery and hospital-level infrastructure appear to be most predictive of malaria control progress (Table 4). This appears well aligned with the Global Fund’s current focus on routine service delivery, including investments in quality improvement as well as infrastructure [44].

In low- and middle-burden countries, health systems factors overall have much lower predictive power for progress in malaria control and moderately more for elimination. In low-burden countries approaching elimination, greater density of lower level health facilities appeared negatively correlated with elimination; however, these results were heavily influenced by China, Mexico and South Korea which all had high coverage of health facilities but did not eliminate malaria and had large reductions in malaria burden (> 99, 94 and 87%, respectively); this may be an indication of more highly developed health systems having stronger surveillance systems and therefore being less likely to have reported zero cases in 2016. In addition, hospital capacity (including density of hospitals and hospital beds) appeared to play a role, likely related to former Soviet countries (Azerbaijan, Tajikistan, Kyrgyzstan) which traditionally had hospital-centred systems and achieved zero indigenous cases of malaria during this time frame. Finally, all models for low-burden countries displayed a negative (but not significant) relationship between governance and malaria elimination, potentially driven by some successful malaria campaigns in countries which scored poorly on governance due to political instability and lack of freedom of expression (e.g., Syria, Iraq, Algeria). This apparent negative relationship with governance is consistent with a detailed case review of nine malaria elimination programmes which found that countries which had clearer lines of accountability and responsibilities (e.g., in Turkey and Turkmenistan) had more successful campaigns [48].

The analysis presented was limited by the quality of data on malaria and health systems in several ways. First, the malaria incidence data analysed depend on local surveillance capacity, and likely do not capture all cases in many countries. This should not affect the main results since the primary outcome analysed was changes over time within countries. Second, the more than 20 publicly available health systems data sources continue to have insufficient coverage for a time-series analysis, which means that period averages have to be used instead of year-specific health system measures. Beyond the inherent challenges in quantitatively disentangling the complex relationship between health systems and other social and ecological factors important for malaria control, the static nature of the data meant that it was not possible to draw causal inference. There is a critical need for better and more consistent data on health systems at the country level which inform on the strength and process of the health system [23, 24]. Several health systems variables that are potentially important for malaria control, such as medicines availability at primary health care centres were entirely excluded due to lack of data coverage. The Service Provision Assessment (SPA) and Service Availability and Readiness Assessment (SARA) surveys present opportunities to better understand country health systems as well as the impact of these factors on malaria control, but are currently only available for 8 and 13 countries, respectively [49, 50]. Higher quality data across countries will allow for additional, more nuanced assessments of health systems readiness for disease control initiatives as well as better monitoring of their health systems impact.

## Conclusions

The results presented in this paper highlight the large heterogeneity in the progress made in malaria control during the period 2000 to 2016, as well as the large heterogeneity in countries’ current health systems infrastructure. While many factors appear relevant for malaria control, the results presented here show that there is no single health systems factor or threshold that dominates other factors in countries’ ability to move towards elimination. Additional health systems strengthening support will likely have the largest impact in high-burden countries, especially in the domain of service delivery.

## Supplementary information

**Additional file 1.** Analytical framework.

**Additional file 2.** Data sources reviewed: references and dates accessed.

**Additional file 3.** Variable coverage.

**Additional file 4.** Full variable definitions for health systems variables.

**Additional file 5.** Descriptive statistics for health systems variables.

**Additional file 6.** Alternate model selection approaches.

**Additional file 7.** Preliminary univariate and adjusted regression analysis.

**Additional file 8.** Preliminary adjusted regression analysis, stratified by initial malaria case burden category in 2000.

**Additional file 9.** Country codes, health system scores, and percent reduction in malaria cases.

**Additional file 10.** Full model selection results with p-values.

## Data Availability

All data used in this analysis are publicly available—full references are provided in the additional files. The compiled dataset analysed for the study is available upon request.

## Abbreviations

DHS: Demographic Health Survey
GDP: Gross domestic product
HDI: Human Development Index
ITN: Insecticide-treated net
WHO: World Health Organization

## Footnotes

1 These countries align with the ‘106 malaria-endemic countries’ detailed in the Lancet Commission on Malaria Eradication with the exception of Kazakhstan [9, 33].

2 Currency is expressed in current international US Dollars, Purchasing Power Parity.
