# Supplementary material for: Health systems and global progress towards malaria elimination, 2000–2016
Source: Malar J. 2020 Apr 8;19:141. doi: 10.1186/s12936-020-03208-6 (PMC7140365; doi:10.1186/s12936-020-03208-6)
Supplement: Supplementary file 1 — Additional file 1. Analytical framework. [file 12936_2020_3208_MOESM1_ESM.docx]

**Additional file 1.** Analytical Framework.


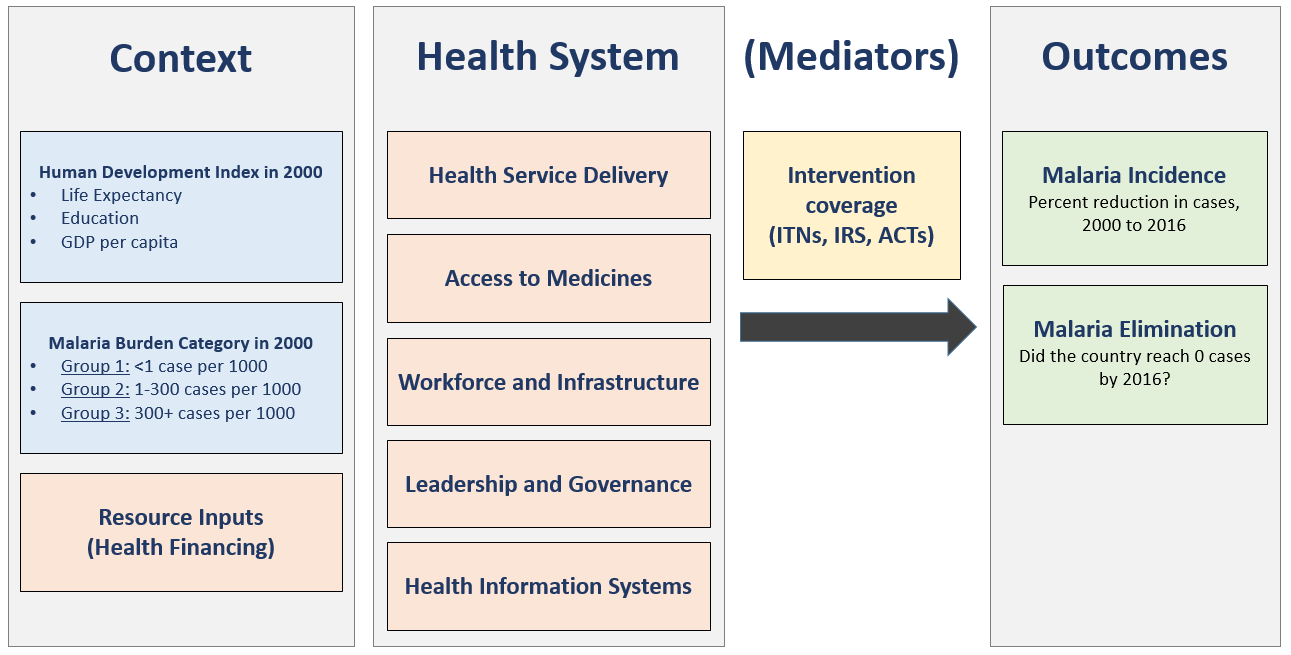


This analytic framework employs the WHO six health system building blocks framework (health service delivery, health financing, health workforce, access to medicines, leadership and governance, and health information systems).[22] In this analysis, health financing is considered a contextual input. Coverage of malaria interventions is considered an output that acts as a mediator on the pathway from health systems inputs to malaria control outcomes, and is not included in final models.
