# Supplementary material for: Health systems and global progress towards malaria elimination, 2000–2016
Source: Malar J. 2020 Apr 8;19:141. doi: 10.1186/s12936-020-03208-6 (PMC7140365; doi:10.1186/s12936-020-03208-6)
Supplement: Supplementary file 2 — Additional file 2. Data sources reviewed: references and dates accessed. [file 12936_2020_3208_MOESM2_ESM.docx]

**Additional file 2.** Data sources reviewed and dates accessed for all databases reviewed.

| # | Database Name | Abbreviation | Citation | Date Accessed | Proposed Usage(s) |
| --- | --- | --- | --- | --- | --- |
| World Health Organization | | | | | |
| 1 | Global Malaria Programme | WHO-GMP | *Personal communication; available from* [1] | 14-Feb-18;  31-Aug-18  3-Sep-18 | Outcome |
| 2 | World Malaria Report | WHO-WMR | [1] | 12-Apr-18 | Outcome/Output |
| 3 | Expanded Program on Immunization | WHO-EPI | [2] | 12-Apr-18 | Predictor |
| 4 | Global Health Observatory | WHO-GHO | [3] | 23-Aug-18 | Predictor |
| World Bank | | | | | |
| 5 | World Development Indicators | WDI | [4] | 12-Apr-18 | Control |
| 6 | World Governance Indicators | WGI | [5] | 11-Apr-18 | Predictor |
| 7 | Logistics Performance Indicators | LPI | [6] | 18-Jul-18 | Predictor |
| 8 | Health, Nutrition and Population Statistics  *(Covers many sources including UN and WHO databases)* | HNP | [7] | 26-Jul-18 | Predictor |
| 9 | World Bank Income Categories | WB | [8] | 6-Aug-18 | Control |
| Malaria Atlas Project | | | | | |
| 10 | Malaria Atlas Project *– General* | MAP | [9] | 2-May-19 | Outcome/Output |
| 11 | Malaria Atlas Project *- Distance to cities database* | MAP | [10] | 19-Jul-18 | Predictor |
| Other variables included in the analysis | | | | | |
| 12 | Demographic Health Survey | DHS | [11] | 5-May-18 | Predictor |
| 13 | Institute for Health Metrics and Evaluation: Development assistance for health | IHME-DAH | [12] | 22-Apr-19 | Supplementary Financing |
| 14 | UN Development Programme:  Human Development Index | UNDP | [13] | 3-Aug-18 | Control |
| 15 | Tatem et al, 2010 | *--* | [14] | 14-Sep-18 | Comparison analysis |
| Other variables extracted and included in final database, but not final analysis | | | | | |
| 16 | Global Fund to Fight AIDS, TB and Malaria | Global Fund | [15] | 19-Jul-18 | Supplementary Financing |
| 17 | Standardized World Income Inequality Database | SWIID | [16] | 11-Apr-18 | Control |
| 18 | Shretta et al, 2017 | *--* | [17] | 11-Oct-18 | Control/Output/Outcome |
| 19 | List of island countries | *--* | [18] | 10-Aug-18 | Control |
| Other data sources considered, but not included in the final database | | | | | |
| 20 | WHO/ Health Action International project on medicine prices and availability | WHO-HAI | [19] | 23-Apr-19 | Not included because not formatted for extraction. |
| 21 | Availability of Generic Medicines | WHO-GHO | [20] | 23-Apr-19 | Not included because data only available for 38 countries. |
| 22 | WHO Service Availability and Readiness Assessment | SARA | [21] | 23-Apr-19 | Not included because data only available for 12 countries. |
| 23 | DHS Service Provision Assessments | SPA | [22] | 23-Apr-19 | Not included because data only available for 12 countries. |

Data Sources Cited

1. World Health Organization, *World Malaria Report*. 2017: Geneva.

2. World Health Organization. *WHO/UNICEF estimates of national immunization coverage*. 2017 [cited 2018 12 April]; Available from: <http://www.who.int/immunization/monitoring_surveillance/routine/coverage/en/index4.html> ].

3. World Health Organization. *Global Health Observatory*. [cited 2018 23 August]; Available from: <http://apps.who.int/gho/data/view.main.30000> ].

4. World Bank. *World Development Indicators database*. [cited 2018 12 April]; Available from: <https://databank.worldbank.org/data/source/world-development-indicators> ].

5. World Bank. *Worldwide Governance Indicators database*. [cited 2018 11 April]; Available from: <https://databank.worldbank.org/data/source/worldwide-governance-indicators> ].

6. World Bank. *Logistics Performance Index*. [cited 2018 18 July]; Available from: <https://lpi.worldbank.org/> ].

7. World Bank. *Health Nutrition and Population Statistics*. [cited 2018 26 July]; Available from: <https://databank.worldbank.org/data/source/health-nutrition-and-population-statistics> ].

8. World Bank. *World Bank Country and Lending Groups*. [cited 2018 6 August]; Available from: <https://datahelpdesk.worldbank.org/knowledgebase/articles/906519-world-bank-country-and-lending-groups> ].

9. Weiss, D.J., et al., *Mapping the global prevalence, incidence, and mortality of Plasmodium falciparum, 2000-17: a spatial and temporal modelling study.* Lancet, 2019.

10. Weiss, D.J., et al., *A global map of travel time to cities to assess inequalities in accessibility in 2015.* Nature, 2018. **553**(7688): p. 333-336.

11. United States Agency for International Development. *Demographic Health Survey*. [cited 2018 5 May]; Available from: <http://www.statcompiler.com> ].

12. Institute for Health Metrics and Evaluation (IHME). *Development Assistance for Health Database 1990-2017*. 2018 [cited 2019 22 April]; Available from: <http://ghdx.healthdata.org/record/ihme-data/development-assistance-health-database-1990-2017> ].

13. United Nations Development Programme (UNDP). *Human Development Reports*. [cited 2018 3 August]; Available from: [http://hdr.undp.org/en/data#](http://hdr.undp.org/en/data) ].

14. Tatem, A.J., et al., *Ranking of elimination feasibility between malaria-endemic countries.* Lancet, 2010. **376**(9752): p. 1579-91.

15. The Global Fund. *Eligibility List 2016*. 5 February 2016 [cited 2018 19 July]; Available from: <https://www.theglobalfund.org/media/5597/core_eligiblecountries2016_list_en.pdf?u=636898877970000000>.

16. Solt, F. *The Standardized World Income Inequality Database (SWIID)*. [cited 2018 11 April]; Version 6.1, October 2017:[Available from: <https://dataverse.harvard.edu/dataset.xhtml?persistentId=hdl:1902.1/11992>.

17. Shretta, R., et al., *Tracking development assistance and government health expenditures for 35 malaria-eliminating countries: 1990-2017.* Malar J, 2017. **16**(1): p. 251.

18. Wikipedia contributors. *List of island countries*. [cited 2018 10 August]; Available from: <https://en.wikipedia.org/wiki/List_of_island_countries>.

19. World Health Organization. *WHO/ Health Action International Project on Medicine Prices and Availability*. [cited 2019 23 April]; Available from: <https://www.who.int/medicines/areas/access/Medicine_Prices_and_Availability/en/>.

20. World Health Organization. *Global Health Observatory data repository: Median availability of selected generic medicines*. [cited 2019 23 April]; Available from: <http://apps.who.int/gho/data/node.main.488?lang=en> ].

21. World Health Organization. *Service Availability and Readiness Assessment (SARA)*. [cited 2019 23 April]; Available from: <https://www.who.int/healthinfo/systems/sara_methods/en/>.

22. United States Agency for International Development. *Service Provision Assessments (SPA)*. The DHS Program: Demograhic and Health Surveys [cited 2019 23 April]; Available from: <https://dhsprogram.com/What-We-Do/Survey-Types/SPA.cfm>.
