# Supplementary material for: Health systems and global progress towards malaria elimination, 2000–2016
Source: Malar J. 2020 Apr 8;19:141. doi: 10.1186/s12936-020-03208-6 (PMC7140365; doi:10.1186/s12936-020-03208-6)
Supplement: Supplementary file 3 — Additional file 3. Variable coverage. [file 12936_2020_3208_MOESM3_ESM.docx]

**Additional file 3.** Coverage for all variables in the final database.

| # | Variable Name | Data Source | | % Data Available (Total)^*^ | Years Available (out of 17,  2000-2016) | % Countries Available (at least one year, out of 105) | Countries available (out of 105) | Included in analysis? [Y/N] | Reason not included (if applicable) |
| --- | --- | --- | --- | --- | --- | --- | --- | --- | --- |
| Malaria Epidemiology [Outcome/Restriction] | | | | | | | | | |
| 1 | Malaria cases per 1000 people | | WHO-GMP/ WDI [1, 2] | 100% | All | 100% | All | Y | -- |
| 2 | Malaria deaths per 1000 people | | WHO-GMP/ WDI [1, 2] | 100% | All | 100% | All | Y | -- |
| 3 | Total malaria population at risk | | WHO-GMP [1] | 96% | All | 96% | 101 | N | Relevance |
| 4 | % Population at risk for malaria | | WHO-GMP/ WDI [1, 2] | 96% | All | 96% | 101 | N | Relevance |
| 5 | Falciparum prevalence rate per 1000 population | | MAP  [3] | 100% | All | 100% | 105 | Y | -- |
| 6 | Malaria programme phase in 2015 | | WHO-GMP [1] | 6% | 2015 only | 97% | 102 | N | Relevance |
| 7 | By 2016, did country ever pursue malaria elimination? | | Shretta et al. [4] | 6% | 2016 only | 100% | All | N | Relevance |
| 8 | In 2016, is country one of 35 malaria-eliminating countries? | | Shretta et al. [4] | 6% | 2016 only | 100% | All | N | Relevance |
| 9 | Is falciparum endemic? (from 2010) | | Tatem et al. [5] | 5% | 2010 only | 92% | 97 | N | Relevance |
| 10 | Is vivax endemic? (from 2010) | | Tatem et al. [5] | 5% | 2010 only | 92% | 97 | N | Relevance |
| Macroeconomic Conditions [Control] | | | | | | | | | |
| 1 | GDP (current US$) | | WDI [2] | 96% | All | 98% | 103 | N | HDI selected as control |
| 2 | GDP growth (annual %) | | WDI [2] | 96% | All | 98% | 103 | N | HDI selected as control |
| 3 | GDP, PPP (current international $) | | WDI [2] | 96% | All | 97% | 102 | N | HDI selected as control |
| 4 | GDP per capita (current US$) | | WDI [2] | 96% | All | 98% | 103 | N | HDI selected as control |
| 5 | GDP per capita growth (annual %) | | WDI [2] | 96% | All | 98% | 103 | N | HDI selected as control |
| 6 | GDP per capita, PPP (current international $) | | WDI [2] | 96% | All | 97% | 102 | N | HDI selected as control |
| 7 | School enrollment, secondary (% net) | | HNP [6] | 42% | All | 85% | 89 | N | HDI selected as control |
| 8 | School enrollment, secondary, female (% net) | | HNP [6] | 42% | All | 85% | 89 | N | HDI selected as control |
| 9 | School enrollment, secondary, male (% net) | | HNP [6] | 42% | All | 85% | 89 | N | HDI selected as control |
| 10 | Human Development Index | | UNDP [7] | 89% | 2000-2015 | 98% | 103 | Y | -- |
| 11 | Income Category (4 categories) | | World Bank [8] | 99% | All | 100% | 105 | Y | -- |
| 12 | Income Category 2 (3 categories) | | World Bank [8] | 99% | All | 100% | 105 | Y | -- |
| 13 | Gini, Disposable Income | | SWIID [9] | 70% | All | 96% | 101 | N | HDI selected as control |
| 14 | Gini, Market Income | | SWIID [9] | 70% | All | 96% | 101 | N | HDI selected as control |
| 15 | Gini, Absolute Redistribution | | SWIID [9] | 22% | All | 24% | 25 | N | Missing data |
| 16 | Gini, Relative Redistribution | | SWIID [9] | 22% | All | 24% | 25 | N | Missing data |
| Demography [Control] | | | | | | | | | |
| 1 | Mortality rate, under-5 (per 1000 live births) | | WDI [2] | 100% | All | 100% | 105 | N | HDI selected as control |
| 2 | Life expectancy at birth, female | | WDI [2] | 94% | 2000-2015 | 100% | 105 | N | HDI selected as control |
| 3 | Life expectancy at birth, total | | WDI [2] | 94% | 2000-2015 | 100% | 105 | N | HDI selected as control |
| 4 | Life expectancy at birth, male | | WDI [2] | 94% | 2000-2015 | 100% | 105 | N | HDI selected as control |
| 5 | Total fertility rate (births per woman) | | WDI [2] | 94% | 2000-2015 | 100% | 105 | N | HDI selected as control |
| 6 | Population growth (annual %) | | WDI [2] | 100% | All | 100% | 105 | N | HDI selected as control |
| 7 | Population, total | | WDI [2] | 100% | All | 100% | 105 | N | HDI selected as control |
| 8 | Urban population growth (annual %) | | WDI [2] | 100% | All | 100% | 105 | N | HDI selected as control |
| 9 | Urban population, total | | WDI [2] | 100% | All | 100% | 105 | N | HDI selected as control |
| 10 | Urban population (% of total) | | WDI [2] | 100% | All | 100% | 105 | N | HDI selected as control |
| 11 | Lifetime risk of maternal death (%) | | HNP [6] | 94% | 2000-2015 | 100% | 105 | N | HDI selected as control |
| 12 | Maternal mortality ratio (modeled estimate, per 100,000 live births) | | HNP [6] | 94% | 2000-2015 | 100% | 105 | N | HDI selected as control |
| 13 | Maternal mortality ratio (national estimate, per 100,000 live births) | | HNP [6] | 22% | All | 97% | 102 | N | HDI selected as control |
| 14 | Net migration | | HNP [6] | 18% | 2002, 2007, 2012 | 100% | 105 | N | HDI selected as control |
| Geography [Control] | | | | | | | | | |
| 1 | WHO Region | | WHO [1] | 100% | All | 100% | 105 | N | HDI selected as control |
| 2 | Land area (sq. km) | | WDI [2] | 99% | All | 99% | 104 | N | HDI selected as control |
| 3 | % of Population living >480 minutes from a city | | MAP [10] | 6% | 2015 | 100% | 105 | N | HDI selected as control |
| 4 | Is country an island? | | *Manual* [11] | 100% | All | 100% | 105 | N | HDI selected as control |
| 5 | Is country an island? (archipelagoes excluded) | | *Manual* [11] | 100% | All | 100% | 105 | N | HDI selected as control |
| Health System Financing [Predictor] | | | | | | | | | |
| 1 | Health expenditure per capita (current US$) | | WDI [2] | 86% | 2000-2014 | 98% | 103 | N | Redundant |
| 2 | Health expenditure per capita, PPP (const 2011 international $) | | WDI [2] | 86% | 2000-2014 | 98% | 103 | Y | -- |
| 3 | Health expenditure, total (% of GDP) | | WDI [2] | 86% | 2000-2014 | 98% | 103 | Y | -- |
| 4 | Health expenditure, public (% total health expenditure) | | WDI [2] | 86% | 2000-2014 | 98% | 103 | Y | -- |
| 5 | Health expenditure, private (% of GDP) | | WDI [2] | 86% | 2000-2014 | 98% | 103 | N | Redundant |
| 6 | Health expenditure, public (% of government expenditure) | | WDI [2] | 85% | 2000-2014 | 98% | 103 | N | Redundant |
| 7 | Health expenditure, public (% of GDP) | | WDI [2] | 86% | 2000-2014 | 98% | 103 | N | Redundant |
| 8 | Impoverishment at the $1.90 Poverty Line (%) | | HNP [6] | 11% | 2000-2015 | 64% | 67 | Preliminary analysis | Relevance |
| 9 | Impoverishment at the $3.10 Poverty Line (%) | | HNP [6] | 11% | 2000-2015 | 64% | 67 | Preliminary analysis | Relevance |
| 10 | Catastrophic Health Expenditure, 10% of total expenditure/income (%) | | HNP [6] | 11% | 2000-2015 | 67% | 70 | Preliminary analysis | Relevance |
| 11 | Catastrophic Health Expenditure, 25% of total expenditure/income (%) | | HNP [6] | 11% | 2000-2015 | 67% | 70 | Preliminary analysis | Relevance |
| 12 | External health expenditure (% total health expenditure) | | HNP [6] | 84% | 2000-2015 | 95% | 100 | Y | -- |
| 13 | Domestic general government health expenditure (% total health expenditure) | | HNP [6] | 90% | 2000-2015 | 98% | 103 | Y | -- |
| 14 | Out-of-pocket expenditure (% total health expenditure) | | HNP [6] | 91% | 2000-2015 | 98% | 103 | Y | -- |
| 15 | Domestic private expenditure (% total health expenditure) | | HNP [6] | 90% | 2000-2015 | 98% | 103 | Y | -- |
| Malaria Financing [Predictor] | | | | | | | | | |
| 1 | Malaria funds, total | | WHO-GMP [1] | 40% | 2010-2016 | 96% | 101 | N | Redundant |
| 2 | Malaria funds per capita | | WHO-GMP [1] | 39% | 2010-2016 | 94% | 99 | Y | -- |
| 3 | Government expenditure on malaria | | WHO-WMR [1] | 12% | 2014-2016 | 80% | 84 | N | Redundant |
| 4 | Foreign expenditure on malaria | | WHO-WMR [1] | 15% | 2014-2016 | 85% | 89 | N | Redundant |
| 5 | Total expenditure on malaria (government + foreign) | | WHO-WMR [1] | 12% | 2014-2016 | 80% | 84 | N | Redundant |
| 6 | Foreign expenditure on malaria (% of total malaria expenditure) | | WHO-WMR [1] | 12% | 2014-2016 | 80% | 84 | Y | -- |
| 7 | DAH to malaria per capita –  All areas | | IHME-DAH/ WDI [2, 12] | 98% | All | 100% | 105 (Imputed) | Preliminary Analysis | Relevance |
| 8 | DAH to malaria per capita – Bednets | | IHME-DAH/ WDI [2, 12] | 98% | All | 100% | 105 (Imputed) | Preliminary Analysis | Relevance |
| 9 | DAH to malaria per capita –  Vector Control | | IHME-DAH/ WDI [2, 12] | 98% | All | 100% | 105 (Imputed) | Preliminary Analysis | Relevance |
| 10 | DAH to malaria per capita – Treatment | | IHME-DAH/ WDI [2, 12] | 98% | All | 100% | 105 (Imputed) | Preliminary Analysis | Relevance |
| 11 | DAH to malaria per capita – Diagnosis | | IHME-DAH/ WDI [2, 12] | 98% | All | 100% | 105 (Imputed) | Preliminary Analysis | Relevance |
| 12 | DAH to malaria per capita – Community Outreach | | IHME-DAH/ WDI [2, 12] | 98% | All | 100% | 105 (Imputed) | Preliminary Analysis | Relevance |
| 13 | DAH to malaria per capita –  Other control | | IHME-DAH/ WDI [2, 12] | 98% | All | 100% | 105 (Imputed) | Preliminary Analysis | Relevance |
| 14 | DAH to malaria per capita –  Health System Strengthening | | IHME-DAH/ WDI [2, 12] | 98% | All | 100% | 105 (Imputed) | Preliminary Analysis | Relevance |
| 15 | DAH to malaria per capita –  Other | | IHME-DAH/ WDI [2, 12] | 98% | All | 100% | 105 (Imputed) | Preliminary Analysis | Relevance |
| 16 | Global Fund eligible in 2016? | | Global Fund  [13] | 6% | 2016 | 100% | 105 | N | Relevance |
| Health Service Delivery [Predictor] | | | | | | | | | |
| 1 | Received third dose of DTP3 vaccine (% children) | | WHO-EPI [14] | 99% | All | 100% | 105 | Y | -- |
| 2 | Immunization, measles (% children ages 12-23 months) | | HNP [6] | 99% | All | 100% | 105 | Y | -- |
| 3 | Pregnant women with at least 4 ANC visits (% of pregnant women) | | HNP [6] | 18% | All | 97% | 102 | Y | -- |
| 4 | Pregnant women receiving prenatal care of at least 1 ANC visit (% of pregnant women) | | HNP [6] | 26% | All | 99% | 104 | N | Redundant |
| 5 | Births attended by skilled health staff (% of total) | | HNP [6] | 39% | All | 100% | 105 | Y | -- |
| 6 | TB treatment success rate (% of new cases) | | HNP [6] | 90% | 2000-2015 | 100% | 105 | Y | -- |
| 7 | TB case detection rate (all forms) | | HNP [6] | 98% | All | 100% | 105 | Y | -- |
| 8 | Universal Health Coverage Index | | HNP [6] | 4% | 2015 | 71% | 75 | N | Redundant |
| 9 | Place of delivery: Health facility | | DHS [15] | 7% | 2000-2013 | 57% | 60 | N | Redundant |
| Malaria Service Delivery [Predictor/ Intermediate Outcome] | | | | | | | | | |
| 1 | % Population protected by IRS | | WHO-GMP [1] | 51% | All | 79% | 83 | Y | -- |
| 2 | ITN coverage (total population) | | WHO-GMP [1] | 70% | All | 91% | 96 | Y | -- |
| 3 | ITN coverage (high risk population) | | WHO-GMP [1] | 63% | All | 78% | 82 | Y | -- |
| 4 | Malaria cases confirmed with RDT/microscopy (%) | | WHO-GMP [1] | 91% | All | 100% | 105 | Y | -- |
| 5 | ITN Access | | WHO-GMP [1] | 38% | All | 38% | 40 | N | Missing data |
| 6 | ITN Use | | WHO-GMP [1] | 38% | All | 38% | 40 | N | Missing data |
| 7 | ITN Gap | | WHO-GMP [1] | 38% | All | 38% | 40 | N | Missing data |
| 8 | ITN coverage (modeled) | | MAP [16] | 41% | All | 41% | 43 | N | Available for Africa only. |
| 9 | % using ITN the previous night | | DHS [15] | 6% | 2003, 2005-2016 | 39% | 41 | N | Missing data |
| 10 | % HHs with at least one ITN | | DHS [15] | 6% | 2002-2016 | 41% | 43 | N | Missing data |
| 11 | % HHs with at least one ITN or IRS in past year | | DHS [15] | 6% | 2002-2016 | 39% | 41 | N | Missing data |
| 12 | % HHs with at least one ITN for every 2 person or IRS in past year | | DHS [15] | 6% | 2002-2016 | 38% | 40 | N | Missing data |
| Access to Medicines [Predictor] | | | | | | | | | |
| 1 | Children under-five with fever receiving antimalarial drugs (%) | | HNP [6] | 13% | All | 65% | 68 | N | Redundant |
| 2 | Children with fever for whom advice/tx was sought from health facility or provider (%) | | DHS [15] | 9% | 2000-2014 | 57% | 60 | Y | -- |
| 3 | Children with fever who took antimalarial drugs (%) | | DHS [15] | 8% | 2000-2014 | 52% | 55 | Y | -- |
| 4 | Children with fever who took antibiotic drugs (%) | | DHS [15] | 6% | 2000-2014 | 52% | 55 | Y | -- |
| 5 | Cough or Fever (for care-seeking vars below) | | DHS/  WHO-GMP [15] | 14% | All | 80% | 84 | N | Not numerical/ Relevance |
| 6 | % fevers/coughs seeking care in public sector | | DHS/  WHO-GMP [15] | 14% | All | 80% | 84 | Preliminary Analysis | Relevance |
| 7 | % fevers/coughs seeking care in private sector | | DHS/  WHO-GMP [15] | 14% | All | 80% | 84 | Preliminary Analysis | Relevance |
| 8 | % fevers/coughs not seeking treatment | | DHS/  WHO-GMP [15] | 14% | All | 80% | 84 | Preliminary Analysis | Relevance |
| Health Workforce [Predictor] | | | | | | | | | |
| 1 | Physicians (per 1000 people) | | HNP [6] | 40% | All | 98% | 103 | Y | -- |
| 2 | Nurses and midwives (per 1000 people) | | HNP [6] | 35% | All | 98% | 103 | Y | -- |
| 3 | CHWs (per 1000 people) | | HNP [6] | 8% | All | 52% | 55 | Y | -- |
| Health Service Capacity [Predictor] | | | | | | | | | |
| 1 | Hospital beds (per 1000 people) | | HNP [6] | 27% | 2000-2012 | 97% | 102 | Y | -- |
| 2 | Health posts (per 1000 people) | | WHO-GHO [17] | 7% | 2010, 2013 | 61% | 64 | Y | -- |
| 3 | Health centres (per 1000 people) | | WHO-GHO [17] | 8% | 2010, 2013 | 65% | 68 | Y | -- |
| 4 | District/Rural Hospitals (per 1000 people) | | WHO-GHO [17] | 8% | 2010, 2013 | 70% | 73 | N | Redundant |
| 5 | Provincial Hospitals (per 1000 people) | | WHO-GHO [17] | 8% | 2010, 2013 | 67% | 70 | N | Redundant |
| 6 | Specialized Hospitals (per 1000 people) | | WHO-GHO [17] | 8% | 2010, 2013 | 70% | 73 | N | Redundant |
| 7 | Hospitals (per 1000 people) | | WHO-GHO [17] | 4% | 2013 | 72% | 76 | Y | -- |
| Governance [Predictor] | | | | | | | | | |
| 1 | Index: Control of corruption | | WGI [18] | 94% | 2000, 2002-2016 | 100% | 105 (Modelled) | Y | -- |
| 2 | Index: Government effectiveness | | WGI [18] | 94% | 2000, 2002-2016 | 100% | 105 (Modelled) | Y | -- |
| 3 | Index: Political stability and absence of violence/terrorism | | WGI [18] | 94% | 2000, 2002-2016 | 100% | 105 (Modelled) | Y | -- |
| 4 | Index: Rule of law | | WGI [18] | 94% | 2000, 2002-2016 | 100% | 105 (Modelled) | Y | -- |
| 5 | Index: Regulatory quality | | WGI [18] | 94% | 2000, 2002-2016 | 100% | 105 (Modelled) | Y | -- |
| 6 | Index: Voice and accountability | | WGI [18] | 94% | 2000, 2002-2016 | 100% | 105 (Modelled) | Y | -- |
| 7 | Index: Logistics performance | | LPI [19] | 25% | 2007, 2010, 2012, 2014, 2016 | 91% | 96 | Y | -- |
| Health Governance [Predictor] | | | | | | | | | |
| 1 | Does country have a National Health Technology Policy guiding use of resources? | | WHO-GHO [17] | 10% | 2010, 2013 | 90% | 95 | N | Relevance |
| 2 | Does country have unit in the MoH responsible for the management of medical devices? | | WHO-GHO [17] | 10% | 2010, 2013 | 90% | 95 | N | Relevance |
| 3 | Compliance with International health regulations (% out of 13) | | WHO-GHO [17] | 29% | 2010-2016 | 100% | 105 | Y | -- |
| Information Systems [Predictor] | | | | | | | | | |
| 1 | Completeness of birth registration (%) | | HNP [6] | 15% | All | 93% | 98 | Y | -- |
| 2 | Completeness of death registration with cause-of-death information (%) | | HNP [6] | 6% | 2002, 2005-2012 | 39% | 41 | N | Missing data |
| 3 | Malaria surveillance report completeness (%) | | WHO-GMP [1] | 99% | All | 99% | 104 | Y | -- |
| Tatem Feasibility Rankings [Comparison Analysis] | | | | | | | | | |
| 1 | Tatem 2010 ranking: technical feasibility, falciparum | | Tatem et al. [5] | 5% | 2010 | 79% | 83 | N | Comparison only |
| 2 | Tatem 2010 ranking: operational feasibility, falciparum | | Tatem et al. [5] | 5% | 2010 | 79% | 83 | N | Comparison only |
| 3 | Tatem 2010 ranking: technical + operational feasibility ranking, falciparum | | Tatem et al. [5] | 5% | 2010 | 79% | 83 | N | Comparison only |
| 4 | Tatem 2010 ranking: operational feasibility, vivax | | Tatem et al. [5] | 5% | 2010 | 86% | 90 | N | Comparison only |
| ***Notes:***  * “% Years Available” is defined as data points available, out of 17 possible years, 2000-2016, and 105 countries (=1785 total) | | | | | | | | | |

Data Sources Cited

1. World Health Organization, *World Malaria Report*. 2017: Geneva.

2. World Bank. *World Development Indicators database*. [cited 2018 12 April]; Available from: <https://databank.worldbank.org/data/source/world-development-indicators> ].

3. Weiss, D.J., et al., *Mapping the global prevalence, incidence, and mortality of Plasmodium falciparum, 2000-17: a spatial and temporal modelling study.* Lancet, 2019.

4. Shretta, R., et al., *Tracking development assistance and government health expenditures for 35 malaria-eliminating countries: 1990-2017.* Malar J, 2017. **16**(1): p. 251.

5. Tatem, A.J., et al., *Ranking of elimination feasibility between malaria-endemic countries.* Lancet, 2010. **376**(9752): p. 1579-91.

6. World Bank. *Health Nutrition and Population Statistics*. [cited 2018 26 July]; Available from: <https://databank.worldbank.org/data/source/health-nutrition-and-population-statistics> ].

7. United Nations Development Programme (UNDP). *Human Development Reports*. [cited 2018 3 August]; Available from: [http://hdr.undp.org/en/data#](http://hdr.undp.org/en/data) ].

8. World Bank. *World Bank Country and Lending Groups*. [cited 2018 6 August]; Available from: <https://datahelpdesk.worldbank.org/knowledgebase/articles/906519-world-bank-country-and-lending-groups> ].

9. Solt, F. *The Standardized World Income Inequality Database (SWIID)*. [cited 2018 11 April]; Version 6.1, October 2017:[Available from: <https://dataverse.harvard.edu/dataset.xhtml?persistentId=hdl:1902.1/11992>.

10. Weiss, D.J., et al., *A global map of travel time to cities to assess inequalities in accessibility in 2015.* Nature, 2018. **553**(7688): p. 333-336.

11. Wikipedia contributors. *List of island countries*. [cited 2018 10 August]; Available from: <https://en.wikipedia.org/wiki/List_of_island_countries>.

12. Institute for Health Metrics and Evaluation (IHME). *Development Assistance for Health Database 1990-2017*. 2018 [cited 2019 22 April]; Available from: <http://ghdx.healthdata.org/record/ihme-data/development-assistance-health-database-1990-2017> ].

13. The Global Fund. *Eligibility List 2016*. 5 February 2016 [cited 2018 19 July]; Available from: <https://www.theglobalfund.org/media/5597/core_eligiblecountries2016_list_en.pdf?u=636898877970000000>.

14. World Health Organization. *WHO/UNICEF estimates of national immunization coverage*. 2017 [cited 2018 12 April]; Available from: <http://www.who.int/immunization/monitoring_surveillance/routine/coverage/en/index4.html> ].

15. United States Agency for International Development. *Demographic Health Survey*. [cited 2018 5 May]; Available from: <http://www.statcompiler.com> ].

16. Malaria Atlas Project. *Plasmodium falciparum parasite rate in 2-10 year olds globally, 2000-2017*. [cited 2019 2 May]; Available from: <https://map.ox.ac.uk/explorer/#/>.

17. World Health Organization. *Global Health Observatory*. [cited 2018 23 August]; Available from: <http://apps.who.int/gho/data/view.main.30000> ].

18. World Bank. *Worldwide Governance Indicators database*. [cited 2018 11 April]; Available from: <https://databank.worldbank.org/data/source/worldwide-governance-indicators> ].

19. World Bank. *Logistics Performance Index*. [cited 2018 18 July]; Available from: <https://lpi.worldbank.org/> ].
