# Supplementary material for: Health systems and global progress towards malaria elimination, 2000–2016
Source: Malar J. 2020 Apr 8;19:141. doi: 10.1186/s12936-020-03208-6 (PMC7140365; doi:10.1186/s12936-020-03208-6)
Supplement: Supplementary file 4 — Additional file 4. Full variable definitions for health systems variables. [file 12936_2020_3208_MOESM4_ESM.docx]

**Additional file 4.** Definitions for all health systems variables included in the preliminary analysis.

| # | Variable Name | Full Definition |
| --- | --- | --- |
| Health Financing | | |
| 1 | Health expenditure per capita, PPP (constant 2011 international $) | Total health expenditure per capita is the sum of public and private health expenditures as a ratio of total population. It covers the provision of health services (preventive and curative), family planning activities, nutrition activities, and emergency aid designated for health but does not include provision of water and sanitation. Data are in international dollars converted using 2011 purchasing power parity (PPP) rates.(1) |
| 2 | Health expenditure, total (% of GDP) | Total health expenditure is the sum of public and private health expenditures as a ratio of total GDP. It covers the provision of health services (preventive and curative), family planning activities, nutrition activities, and emergency aid designated for health but does not include provision of water and sanitation.(1) |
| 3 | Health expenditure, public (% total health expenditure) | Public health expenditure consists of recurrent and capital spending from government (central and local) budgets, external borrowings and grants (including donations from international agencies and nongovernmental organizations), and social (or compulsory) health insurance funds. (1) |
| 4 | Impoverishment at the $1.90 Poverty Line (%) | Proportion of population pushed below the $1.90 ($ 2011 PPP) poverty line by out-of-pocket health care expenditure, expressed as a percentage of a total population of a country.(1) |
| 5 | Impoverishment at the $3.10 Poverty Line (%) | Proportion of population pushed below the $3.10 ($ 2011 PPP) poverty line by out-of-pocket health care expenditure, expressed as a percentage of a total population of a country.(1) |
| 6 | Catastrophic Health Expenditure, 10% of total expenditure/income (%) | Proportion of population spending more than 10% of household consumption or income on out-of-pocket health care expenditure, expressed as a percentage of a total population of a country. (1) |
| 7 | Catastrophic Health Expenditure, 25% of total expenditure/income (%) | Proportion of population spending more than 25% of household consumption or income on out-of-pocket health care expenditure, expressed as a percentage of a total population of a country. (1) |
| 8 | External health expenditure (% total health expenditure) | Share of current health expenditures funded from external sources. External sources compose of direct foreign transfers and foreign transfers distributed by government encompassing all financial inflows into the national health system from outside the country. External sources either flow through the government scheme or are channeled through non-governmental organizations or other schemes.(1) |
| 9 | Domestic general government health expenditure (% total health expenditure) | Share of current health expenditures funded from domestic public sources for health. Domestic public sources include domestic revenue as internal transfers and grants, transfers, subsidies to voluntary health insurance beneficiaries, non-profit institutions serving households (NPISH) or enterprise financing schemes as well as compulsory prepayment and social health insurance contributions. They do not include external resources spent by governments on health.(1) |
| 10 | Out-of-pocket expenditure (% total health expenditure) | Share of out-of-pocket payments of total current health expenditures. Out-of-pocket payments are spending on health directly out-of-pocket by households.(1) |
| 11 | Domestic private expenditure (% total health expenditure) | Private health expenditure includes direct household (out-of-pocket) spending, private insurance, charitable donations, and direct service payments by private corporations.(1) |
| Malaria Financing | | |
| 12 | Malaria funds per capita | Estimated funding for malaria (domestic and external) per person at risk of malaria (Methods and sources described in WMR 2017).(2) |
| 13 | Foreign expenditure on malaria (% of total malaria expenditure) | Percentage of external funding for malaria out of total funding for malaria (Methods and sources described in WMR 2017).(2) |
| 14 | DAH to malaria per capita – All areas | Funds for health disbursed from source to channel to recipient country for malaria, disaggregated by community outreach, bednets, indoor spraying, other control, diagnosis, health systems strengthening, treatment, and other.(3) |
| 15 | DAH to malaria per capita – Bednets |  |
| 16 | DAH to malaria per capita – Vector Control |  |
| 17 | DAH to malaria per capita – Treatment |  |
| 18 | DAH to malaria per capita – Diagnosis |  |
| 19 | DAH to malaria per capita – Community Outreach |  |
| 20 | DAH to malaria per capita – Other control |  |
| 21 | DAH to malaria per capita – Health System Strengthening |  |
| 22 | DAH to malaria per capita – Other |  |
| Health Service Delivery | | |
| 23 | Received third dose of DTP3 vaccine (% children) | Child immunization, DPT, measures the percentage of children ages 12-23 months who received DPT vaccinations before 12 months or at any time before the survey. A child is considered adequately immunized against diphtheria, pertussis (or whooping cough), and tetanus (DPT) after receiving three doses of vaccine.(4) |
| 24 | Immunization, measles (% children) | Child immunization, measles, measures the percentage of children ages 12-23 months who received the measles vaccination before 12 months or at any time before the survey. A child is considered adequately immunized against measles after receiving one dose of vaccine.(5) |
| 25 | Pregnant women with at least 4 ANC visits (% of pregnant women) | Pregnant women receiving prenatal care, at least four times, are the percentage of women attended at least four times during pregnancy by skilled health personnel for reasons related to pregnancy.(5) |
| 26 | Births attended by skilled health staff (% of total) | Births attended by skilled health staff are the percentage of deliveries attended by personnel trained to give the necessary supervision, care, and advice to women during pregnancy, labor, and the postpartum period; to conduct deliveries on their own; and to care for newborns.(6) |
| 27 | TB treatment success rate (% of new cases) | Tuberculosis treatment success rate is the percentage of all new tuberculosis cases (or new and relapse cases for some countries) registered under a national tuberculosis control programme in a given year that successfully completed treatment, with or without bacteriological evidence of success ("cured" and "treatment completed" respectively).(5) |
| 28 | TB case detection rate (all forms) | Tuberculosis case detection rate (all forms) is the number of new and relapse tuberculosis cases notified to WHO in a given year, divided by WHO's estimate of the number of incident tuberculosis cases for the same year, expressed as a percentage.(5) |
| Malaria Service Delivery | | |
| 29 | % Population protected by IRS | Percentage of population at risk of malaria protected by IRS. Most IRS in malaria endemic countries is delivered in focal areas, but for consistency of trend, all population at risk is used as denominator.(2) |
| 30 | ITN coverage (total population) | Percentage of all population at risk who slept under a bed net before the night of survey. Methods described in the 2017 World Malaria Report.(2) |
| 31 | ITN coverage (high risk population) | Percentage of all population at high risk who slept under a bed net before the night of survey. Methods described in 2017 World Malaria Report.(2) |
| 32 | Malaria cases confirmed with RDT/microscopy (%) | Percentage of suspected malaria cases in the public health sector who were tested for malaria using RDTs or microscopy.(2) |
| Access to Medicines | | |
| 33 | Children with fever for whom advice/tx was sought from health facility or provider (%) | Percentage of children with fever in the two weeks preceding the survey for whom advice or treatment was sought from a health facility or provider.(6) |
| 34 | Children with fever who took antimalarial drugs (%) | Percentage of children with fever in the two weeks preceding the survey who took antimalarial drugs.(6) |
| 35 | Children with fever who took antibiotic drugs (%) | Percentage of children with fever in the two weeks preceding the survey who took antibiotic drugs.(6) |
| 36 | % fevers/coughs seeking care in public sector | Percentage of children with fever/cough in the two weeks preceding the survey who sought care in the public sector (Household survey data summarized the 2017 World Malaria Report).(2) |
| 37 | % fevers/coughs seeking care in private sector | Percentage of children with fever/cough in the two weeks preceding the survey who sought care in the private sector (Household survey data summarized the 2017 World Malaria Report).(2) |
| 38 | % fevers/coughs not seeking treatment | Percentage of children with fever/cough in the two weeks preceding the survey who sought no care (Household survey data summarized the 2017 World Malaria Report).(2) |
| Health Workforce and Capacity | | |
| 39 | Physicians (per 1000 people) | Physicians include generalist and specialist medical practitioners.(5) |
| 40 | Nurses and midwives (per 1000 people) | Nurses and midwives include professional nurses, professional midwives, auxiliary nurses, auxiliary midwives, enrolled nurses, enrolled midwives and other associated personnel, such as dental nurses and primary care nurses.(5) |
| 41 | CHWs (per 1000 people) | Community health workers include various types of community health aides, many with country-specific occupational titles such as community health officers, community health-education workers, family health workers, lady health visitors and health extension package.(5) |
| 42 | Hospital beds (per 1000 people) | Hospital beds include inpatient beds available in public, private, general, and specialized hospitals and rehabilitation centers. In most cases beds for both acute and chronic care are included.(5) |
| 43 | Health posts (per 1000 people) | Number of health posts from the public and private sectors, normalized to per 1000 population. Health posts are either community centres or health environments with a very limited number of beds with limited curative and preventive care resources normally assisted by health workers or nurses.(7) |
| 44 | Health centres (per 1000 people) | Number of health centres from the public and private sectors, normalized to per 1000 population. (7) |
| 45 | Hospitals (per 1000 people) | Number of hospitals from the public and private sectors, normalized to per 1000 population.(7) |
| Governance | | |
| 46 | Index: Control of corruption | Control of Corruption captures perceptions of the extent to which public power is exercised for private gain, including both petty and grand forms of corruption, as well as "capture" of the state by elites and private interests. Estimate gives the country's score on the aggregate indicator, in units of a standard normal distribution, i.e. ranging from approximately -2.5 to 2.5. (8) |
| 47 | Index: Government effectiveness | Government Effectiveness captures perceptions of the quality of public services, the quality of the civil service and the degree of its independence from political pressures, the quality of policy formulation and implementation, and the credibility of the government's commitment to such policies. Estimate gives the country's score on the aggregate indicator, in units of a standard normal distribution, i.e. ranging from approximately -2.5 to 2.5. (8) |
| 48 | Index: Political stability and absence of violence/terrorism | Political Stability and Absence of Violence/Terrorism measures perceptions of the likelihood of political instability and/or politically-motivated violence, including terrorism. Estimate gives the country's score on the aggregate indicator, in units of a standard normal distribution, i.e. ranging from approximately -2.5 to 2.5. (8) |
| 49 | Index: Rule of law | Rule of Law captures perceptions of the extent to which agents have confidence in and abide by the rules of society, and in particular the quality of contract enforcement, property rights, the police, and the courts, as well as the likelihood of crime and violence. Estimate gives the country's score on the aggregate indicator, in units of a standard normal distribution, i.e. ranging from approximately -2.5 to 2.5. (8) |
| 50 | Index: Regulatory quality | Regulatory Quality captures perceptions of the ability of the government to formulate and implement sound policies and regulations that permit and promote private sector development. Estimate gives the country's score on the aggregate indicator, in units of a standard normal distribution, i.e. ranging from approximately -2.5 to 2.5. (8) |
| 51 | Index: Voice and accountability | Voice and Accountability captures perceptions of the extent to which a country's citizens are able to participate in selecting their government, as well as freedom of expression, freedom of association, and a free media. Estimate gives the country's score on the aggregate indicator, in units of a standard normal distribution, i.e. ranging from approximately -2.5 to 2.5. (8) |
| 52 | Index: Logistics performance | Summary indicator of logistics sector performance based on 6 components: (1) efficiency of customs and border clearance, (2) quality of trade and transport infrastructure, (3) ease of arranging competitively priced shipments, (4) competence and quality of logistics services , (5) ability to track and trace consignments, and (6) frequency with which shipments reach consignees within expected delivery times; rated on a scale of 1-5 with 5 being the highest. (8) |
| 53 | Compliance with International health regulations (% out of 13) | Percentage of attributes of 13 core capacities that have been attained at a specific point in time. The 13 core capacities are: (1) National legislation, policy and financing; (2) Coordination and National Focal Point communications; (3) Surveillance; (4) Response; (5) Preparedness; (6) Risk communication; (7) Human resources; (8) Laboratory; (9) Points of entry; (10) Zoonotic events; (11) Food safety; (12) Chemical events; (13) Radionuclear emergencies.(7) |
| Information Systems | | |
| 54 | Completeness of birth registration (%) | Completeness of birth registration is the percentage of children under age 5 whose births were registered at the time of the survey. The numerator of completeness of birth registration includes children whose birth certificate was seen by the interviewer or whose mother or caretaker says the birth has been registered. [4] |
| 55 | Malaria surveillance report completeness (%) | Percentage of expected reports in the public health in a year received by the central health management and information systems or the NMCPs as reported by countries.(2) |

**Data Sources Cited**

1. World Bank. World Development Indicators Database [Internet]. [cited 2018 Apr 12]. Available from: https://databank.worldbank.org/data/source/world-development-indicators

2. World Health Organization. World malaria report 2017. Geneva: World Health Organization; 2017.

3. Institute for Health Metrics and Evaluation. Development Assistance for Health Database 1990-2017 [Internet]. 2017 [cited 2019 Apr 22]. Available from: http://ghdx.healthdata.org/record/ihme-data/development-assistance-health-database-1990-2017

4. World Health Organization. WHO/UNICEF estimates of national immunization coverage [Internet]. 2017 [cited 2018 Apr 12]. Available from: http://www.who.int/immunization/monitoring_surveillance/routine/coverage/en/index4.html

5. World Bank. Health Nutrition and Population Statistics [Internet]. [cited 2018 Jul 26]. Available from: https://databank.worldbank.org/data/source/health-nutrition-and-population-statistics

6. United States Agency for International Development. Demographic Health Survey. [Internet]. [cited 2018 May 5]. Available from: http://www.statcompiler.com

7. World Health Organization. Global Health Observatory [Internet]. [cited 2018 Aug 23]. Available from: http://apps.who.int/gho/data/view.main.30000

8. World Bank. Worldwide Governance Indicators Database [Internet]. 2018. Available from: https://databank.worldbank.org/data/source/worldwide-governance-indicators
