# Supplementary material for: Health systems and global progress towards malaria elimination, 2000–2016
Source: Malar J. 2020 Apr 8;19:141. doi: 10.1186/s12936-020-03208-6 (PMC7140365; doi:10.1186/s12936-020-03208-6)
Supplement: Supplementary file 5 — Additional file 5. Descriptive statistics for health systems variables. [file 12936_2020_3208_MOESM5_ESM.docx]

**Additional file 5.** Descriptive statistics for all health systems variables entering the Principal Component Analysis.

| **No.** | **Variable** | **Baseline (2000)**  **[Original Dataset]** | | **Endline (2016)**  **[Original Dataset]** | | **Average (2000-2016)**  **[Original dataset]** | | **Average (2000-2016)**  **[Imputed dataset]** | |
| --- | --- | --- | --- | --- | --- | --- | --- | --- | --- |
|  |  | **Mean** | **95% CI** | **Mean** | **95% CI** | **Mean** | **95% CI** | **Mean** | **95% CI** |
| **Health service delivery, routine services** | |  |  |  |  |  |  |  |  |
| 1 | Received third dose of DTP3 vaccine (% children) | 72.806 | (68.667, 76.945) | 84.324 | (81.167, 87.481) | 80.726 | (79.912, 81.541) | *[Same as original]* | |
| 2 | Immunization, measles (% children ages 12-23 months) | 73.359 | (69.337, 77.381) | 83.610 | (80.443, 86.776) | 80.209 | (79.405, 81.013) | *[Same as original]* | |
| 3 | Pregnant women with at least 4 ANC visits (% pregnant women) | 47.702 | (40.495, 54.909) | 68.511 | (58.913, 78.108) | 60.284 | (57.688, 62.880) | 61.685 | (57.338, 66.032) |
| 4 | Births attended by skilled health staff (% of total) | 65.004 | (58.016, 71.992) | 79.600 | (66.060, 93.140) | 76.238 | (74.312, 78.163) | *[Same as original]* | |
| 5 | TB treatment success rate (% of new cases) | 75.118 | (72.739, 77.497) | -- | -- | 78.598 | (78.009, 79.187) | *[Same as original]* | |
| 6 | TB case detection rate (all forms) | 56.654 | (51.503, 61.804) | 67.343 | (64.289, 70.397) | 62.865 | (61.922, 63.809) | *[Same as original]* | |
| **Access to medicines** | |  |  |  |  |  |  |  |  |
| 1 | DHS: Children with fever for whom advice/tx was sought from health facility or provider (%) | 43.536 | (38.538, 48.535) | -- | -- | 52.450 | (50.039, 54.861) | 53.070 | (50.728, 55.412) |
| 2 | DHS: Children with fever who took antibiotic drugs (%) | -- | -- | -- | -- | 30.704 | (28.091, 33.318) | 34.142 | (31.649, 36.634) |
| **Health workforce** | |  |  |  |  |  |  |  |  |
| 1 | Physicians (per 1000 people) | 1.044 | (0.728, 1.360) | 0.676 | (0.208, 1.143) | 0.971 | (0.894, 1.049) | 0.756 | (0.577, 0.935) |
| 2 | Nurses and midwives (per 1000 people) | 1.719 | (1.334, 2.103) | 2.398 | (0.935, 3.861) | 1.997 | (1.833, 2.160) | 1.693 | (1.345, 2.040) |
| 3 | CHWs (per 1000 people) | 0.480 | (0.074, 0.885) | 0.292 | (-0.185, 0.768) | 0.365 | (0.296, 0.434) | 0.520 | (0.380, 0.661) |
| **Health system capacity: basic health centres** | |  |  |  |  |  |  |  |  |
| 1 | Health posts (per 1000 people) | -- | -- | -- | -- | 0.191 | (0.124, 0.258) | 0.237 | (0.147, 0.327) |
| 2 | Health centres (per 1000 people) | -- | -- | -- | -- | 0.053 | (0.039, 0.068) | 0.057 | (0.038, 0.076) |
| **Health system capacity: hospital capacity** | |  |  |  |  |  |  |  |  |
| 1 | Hospital beds (per 1000 people) | 3.674 | (2.521, 4.827) | -- | -- | 2.147 | (1.971, 2.322) | 1.856 | (1.496, 2.216) |
| 2 | Hospitals (per 1000 people) | -- | -- | -- | -- | 0.020 | (0.006, 0.035) | 0.019 | (0.008, 0.030) |
| **Governance** | |  |  |  |  |  |  |  |  |
| 1 | Index: Control of corruption | -0.602 | (-0.719, -0.486) | -0.648 | (-0.766, -0.531) | -0.641 | (-0.669, -0.614) | *[Same as original]* | |
| 2 | Index: Government effectiveness | -0.593 | (-0.717, -0.470) | -0.634 | (-0.763, -0.504) | -0.631 | (-0.661, -0.601) | *[Same as original]* | |
| 3 | Index: Political stability and absence of violence/terrorism | -0.527 | (-0.696, -0.358) | -0.590 | (-0.756, -0.424) | -0.606 | (-0.648, -0.565) | *[Same as original]* | |
| 4 | Index: Rule of law | -0.646 | (-0.773, -0.518) | -0.663 | (-0.791, -0.536) | -0.686 | (-0.716, -0.657) | *[Same as original]* | |
| 5 | Index: Regulatory quality | -0.553 | (-0.695, -0.411) | -0.624 | (-0.756, -0.491) | -0.616 | (-0.649, -0.583) | *[Same as original]* | |
| 6 | Index: Voice and accountability | -0.570 | (-0.721, -0.418) | -0.549 | (-0.708, -0.391) | -0.596 | (-0.634, -0.559) | *[Same as original]* | |
| 7 | Index: Logistics performance | -- | -- | 2.597 | (2.502, 2.692) | 2.577 | (2.539, 2.615) | 2.560 | (2.493, 2.626) |
| 8 | Compliance with International Health Regulations | -- | -- | 73.065 | (68.033, 78.096) | 63.654 | (61.677, 65.630) | *[Same as original]* | |
| **Health information systems** | |  |  |  |  |  |  |  |  |
| 1 | Completeness birth registration (%) | 71.178 | (48.121, 94.234) | 67.060 | (41.105, 93.015) | 68.601 | (65.244, 71.958) | 70.898 | (65.612, 76.184) |
| 2 | Malaria surveillance report completeness (%) | 0.879 | (0.849, 0.910) | 0.909 | (0.881, 0.936) | 0.886 | (0.878, 0.893) | 0.885 | (0.860, 0.911) |
