# Supplementary material for: Health systems and global progress towards malaria elimination, 2000–2016
Source: Malar J. 2020 Apr 8;19:141. doi: 10.1186/s12936-020-03208-6 (PMC7140365; doi:10.1186/s12936-020-03208-6)
Supplement: Supplementary file 6 — Additional file 6. Alternate model selection approaches. [file 12936_2020_3208_MOESM6_ESM.docx]

**Additional file 6.** Alternate approaches.

Some additional model selection methods were considered to develop models using single health systems variables: forward and forward stepwise selection, backward and backward stepwise selection, least absolute shrinkage and selection operator (LASSO), as well as systematic variable grid searches with fixed model size (not shown here). Overall, LASSO and backward stepwise models performed best in terms of root mean-squared error as well as model fit, though LASSO selected nearly all variables and therefore was susceptible to overfitting. Thus, we preferred the more parsimonious backwards stepwise models for our final analysis.

R-squared from best performing models for reduction in malaria cases between 2000 and 2016 are presented below. All regressions adjusted for initial HDI and malaria case burden group in 2000. 40 health systems variables were included for model selection.^†^

| **#** | **Selection Method** | **Definition** | **2000-2016** |
| --- | --- | --- | --- |
| 1 | Forward Selection | Add terms with p<.1 | R^2^ = .1612 |
| 2 | Forward Stepwise | Add terms with p<.1; remove terms p>.2 | R^2^ = .1612 |
| 3 | Backward Selection | Remove terms with p>.1 | R^2^ = .2868 |
| 4 | Backward Stepwise | Remove terms with p>.2; add terms p<.1 | R^2^ = .3780 |
| 5 | Backward Stepwise, excluding outliers* | Remove terms with p>.2; add terms p<.1 | R^2^ = .5631 |
| 6 | LASSO | Least absolute shrinkage and selection operator | R^2^ = .4388 |
| ***Notes:***  ^†^ Overall 40 health systems variables (39 unique variables plus 1 log variable) were included in the model search; due to data availability, we used period averages of the health systems variables for the 2000-2016 period.  *Outliers here are Venezuela and Rwanda, which both experienced large increases in cases. | | | |
