# Supplementary material for: Health systems and global progress towards malaria elimination, 2000–2016
Source: Malar J. 2020 Apr 8;19:141. doi: 10.1186/s12936-020-03208-6 (PMC7140365; doi:10.1186/s12936-020-03208-6)
Supplement: Supplementary file 8 — Additional file 8. Preliminary adjusted regression analysis, stratified by initial malaria case burden category in 2000. [file 12936_2020_3208_MOESM8_ESM.docx]

**Additional file 8.** Adjusted regression analysis for progress in malaria control between 2000 and 2016, using outcome of reduction in malaria cases between 2000 and 2016, and stratified by initial malaria case burden category in 2000. All regressions are adjusted for initial HDI in 2000.

Regression analysis was performed prior to imputing missing data, and all regressions exclude Venezuela, which was an outlier due to political instability.

| No. | Variable Name | % Reduction in Malaria Cases, Adjusted  Low-Burden Countries (<1 case per 1000 in 2000) | | | % Reduction in Malaria Cases, Adjusted  Middle-Burden Countries (1-300 cases per 1000 in 2000) | | | % Reduction in Malaria Cases, Adjusted  High-Burden Countries (300+ cases per 1000 in 2000) | | |
| --- | --- | --- | --- | --- | --- | --- | --- | --- | --- | --- |
|  |  | **β** | **95% CI** | **p** | **β** | **95% CI** | **p** | **β** | **95% CI** | **p** |
| Health System Financing | |  | | |  | | |  | | |
| 1 | Health expenditure per capita, PPP | -0.009 | -0.052,0.035 | 0.692 | 0.023 | -0.042,0.088 | 0.485 | -0.04 | -0.109,0.028 | 0.236 |
| 2 | Health expenditure (% of GDP) | -2.122 | -7.937,3.693 | 0.459 | -0.223 | -0.876,0.430 | 0.495 | 2.475 | -2.752,7.702 | 0.337 |
| 3 | Health expenditure, public (% total health expenditure) | -0.030 | -0.604,0.544 | 0.915 | 5.177 | -10.684,21.037 | 0.508 | 0.162 | -0.413,0.738 | 0.564 |
| 4 | External health expenditure (% total health expenditure) | -0.200 | -4.382,3.982 | 0.922 | -0.049 | -0.758,0.661 | 0.891 | **0.806*** | 0.150,1.463 | 0.018 |
| 5 | Domestic government expenditure (% total health expenditure) | 0.025 | -0.542,0.592 | 0.929 | 0.241 | -0.371,0.854 | 0.432 | 0.676 | -0.157,1.509 | 0.107 |
| 6 | Domestic private expenditure (% total health expenditure) | -0.014 | -0.589,0.560 | 0.960 | 0.176 | -0.388,0.740 | 0.533 | -0.600* | -1.054,-0.146 | 0.012 |
| 7 | Out-of-pocket expenditure (% total health expenditure) | 0.047 | -0.458,0.552 | 0.850 | 0.023 | -0.042,0.088 | 0.485 | **-0.746**** | -1.175,-0.317 | 0.002 |
| Malaria Financing | |  | | |  | | |  | | |
| 1 | Malaria funds per capita | 0.055 | -0.099,0.210 | 0.462 | 1.188 | -0.469,2.844 | 0.156 | 1.055 | -5.039,7.148 | 0.723 |
| 2 | Foreign expenditure on malaria (% of total malaria expenditure) | 0.441 | -1.522,2.403 | 0.628 | -0.023 | -0.790,0.743 | 0.951 | 0.248 | -0.663,1.158 | 0.577 |
| 3 | DAH to malaria per capita –  All areas (in thousands of USD) | -35.664 | -95.511,24.183 | 0.231 | 0.049 | -6.693,6.790 | 0.988 | **9.693**** | 3.145,16.241 | 0.006 |
| 4 | DAH to malaria per capita –  Bednets (in thousands of USD) | -195.514 | -560.442, 169.414 | 0.280 | 4.157 | -24.893,33.208 | 0.775 | **80.678***** | 38.225,123.130 | 0.001 |
| 5 | DAH to malaria per capita –  Vector Control (in thousands of USD) | -1.40E+03 | -3.8e+03, 1005.895 | 0.241 | -7.856 | -75.494,59.781 | 0.816 | 132.443 | -11.906, 276.793 | 0.070 |
| 6 | DAH to malaria per capita – Treatment (in thousands of USD) | -169.403 | -488.424, 149.617 | 0.285 | -10.747 | -53.874,32.380 | 0.619 | **46.083*** | 2.884,89.281 | 0.038 |
| 7 | DAH to malaria per capita – Diagnosis (in thousands of USD) | -281.596 | -799.615, 236.423 | 0.274 | 29.468 | -51.081,110.017 | 0.466 | **214.642*** | 43.628,385.656 | 0.016 |
| 8 | DAH to malaria per capita – Community Outreach (in thousands of USD) | -1.90E+03 | -4.7e+03, 901.008 | 0.175 | 8.642 | -32.420,49.704 | 0.674 | 34.868 | -0.934,70.669 | 0.056 |
| 9 | DAH to malaria per capita –  Other control (in thousands of USD) | -246.232 | -686.485, 194.020 | 0.260 | **-81.470*** | -157.958,-4.982 | 0.037 | 39.357 | -22.261, 100.975 | 0.199 |
| 10 | DAH to malaria per capita –  Health System Strengthening (in thousands of USD) | -118.17 | -324.235, 87.896 | 0.249 | 9.199 | -19.992,38.391 | 0.529 | **50.586*** | 7.381,93.791 | 0.024 |
| 11 | DAH to malaria per capita –  Other (in thousands of USD) | -201.519 | -624.896, 221.858 | 0.336 | -11.718 | -64.811,41.374 | 0.659 | **21.337*** | 2.160,40.514 | 0.031 |
| Health Service Delivery | |  | | |  | | |  | | |
| 1 | Received third dose of DTP3 vaccine (% children) | 0.035 | -1.138,1.207 | 0.952 | 0.114 | -0.677,0.905 | 0.774 | **0.812**** | 0.289,1.335 | 0.004 |
| 2 | Immunization, measles (% children ages 12-23 months) | -0.109 | -1.264,1.046 | 0.847 | 0.173 | -0.592,0.938 | 0.651 | **1.030**** | 0.462,1.598 | 0.001 |
| 3 | Pregnant women with at least 4 ANC visits (% of pregnant women) | -0.118 | -0.876,0.640 | 0.749 | 0.398 | -0.184,0.980 | 0.176 | 0.554 | -0.155,1.262 | 0.119 |
| 4 | Births attended by skilled health staff (% of total) | 0.418 | -0.350,1.185 | 0.273 | 0.218 | -0.303,0.738 | 0.404 | 0.334 | -0.368,1.036 | 0.334 |
| 5 | TB treatment success rate (% of new cases) | 0.439 | -0.529,1.406 | 0.360 | 0.713 | -0.413,1.839 | 0.209 | 1.143 | -0.051,2.338 | 0.060 |
| 6 | TB case detection rate (all forms) | 1.055* | 0.168,1.941 | 0.022 | 0.096 | -0.565,0.758 | 0.771 | 0.340 | -0.382,1.061 | 0.339 |
| 7 | Population protected by IRS | -0.112 | -0.672,0.448 | 0.662 | 0.52 | -0.393,1.434 | 0.257 | **0.993*** | 0.004,1.981 | 0.049 |
| 8 | ITN coverage (total population) | 0.005 | -0.013,0.022 | 0.564 | 0.106 | -0.171,0.384 | 0.445 | **0.701**** | 0.278,1.125 | 0.002 |
| 9 | ITN coverage (high risk population) | 0.055 | -0.083,0.192 | 0.369 | **0.145*** | 0.009,0.282 | 0.038 | **0.757**** | 0.263,1.250 | 0.004 |
| 10 | Malaria cases confirmed with RDT/microscopy (%) | 0.03 | -0.469,0.529 | 0.903 | 0.079 | -0.322,0.481 | 0.693 | 0.119 | -0.485,0.723 | 0.687 |
| Access to Medicines | |  | | |  | | |  | | |
| 1 | DHS: Children with fever for whom advice/tx was sought from health facility or provider (%) | -2.361 | -4.808,0.086 | 0.056 | 0.708 | -0.785,2.201 | 0.339 | **0.839*** | 0.046,1.631 | 0.039 |
| 2 | DHS: Children with fever who took antimalarial drugs (%) | -2.362 | -23.488,18.764 | 0.678 | -1.048 | -2.280,0.184 | 0.092 | 0.125 | -0.624,0.874 | 0.730 |
| 3 | DHS: Children with fever who took antibiotic drugs (%) | -0.734 | -3.357,1.890 | 0.481 | 0.454 | -0.970,1.879 | 0.517 | 0.725 | -0.131,1.580 | 0.092 |
| 4 | Fevers/coughs seeking care in public sector (%) | 0.357 | -0.429,1.143 | 0.348 | 0.696 | -0.116,1.507 | 0.091 | 0.791 | -0.042,1.625 | 0.062 |
| 5 | Fevers/coughs seeking care in private sector (%) | -0.276 | -0.910,0.358 | 0.368 | -0.174 | -0.857,0.509 | 0.609 | -0.115 | -0.972,0.743 | 0.783 |
| 6 | Fevers/coughs not seeking treatment (%) | 0.139 | -0.806,1.084 | 0.758 | -0.443 | -1.347,0.461 | 0.328 | -0.794 | -1.776,0.189 | 0.107 |
| Health Workforce and Capacity | |  | | |  | | |  | | |
| 1 | Physicians (per 1000 people) | 4.113 | -5.605,13.831 | 0.392 | 14.098 | -5.882,34.078 | 0.162 | -57.43 | -214.644, 99.784 | 0.457 |
| 2 | Nurses and midwives (per 1000 people) | 1.294 | -2.414,5.003 | 0.479 | **11.504*** | 0.772,22.237 | 0.036 | 3.174 | -14.067,20.415 | 0.706 |
| 3 | CHWs (per 1000 people) | -8.126 | -22.224,5.971 | 0.220 | -32.659 | -81.102,15.785 | 0.176 | **33.285*** | 5.356,61.213 | 0.023 |
| 4 | Hospital beds (per 1000 people) | 1.776 | -3.389,6.942 | 0.485 | 4.243 | -0.968,9.454 | 0.108 | 9.772 | -8.886,28.430 | 0.289 |
| 5 | Health posts (per 1000 people) | 9.158 | -55.687,74.002 | 0.765 | 5.896 | -18.288,30.079 | 0.623 | -55.009 | -229.822, 119.805 | 0.499 |
| 6 | Health centres (per 1000 people) | -19.911 | -173.692, 133.870 | 0.781 | -43.842 | -295.161,207.477 | 0.725 | 79.208 | -90.162, 248.579 | 0.335 |
| 7 | Hospitals (per 1000 people) | 348.213 | -365.825, 1062.251 | 0.317 | 87.154 | -689.737,864.045 | 0.821 | 71.008 | -27.517, 169.532 | 0.145 |
| Governance | |  | | |  | | |  | | |
| 1 | Index: Control of corruption | -4.425 | -21.845,12.996 | 0.605 | -4.536 | -24.160,15.087 | 0.644 | 3.087 | -26.248,32.422 | 0.829 |
| 2 | Index: Government effectiveness | -2.561 | -21.823,16.702 | 0.787 | -1.189 | -19.995,17.616 | 0.899 | -0.306 | -27.019,26.407 | 0.981 |
| 3 | Index: Political stability and absence of violence/terrorism | -4.55 | -18.543,9.443 | 0.509 | 7.955 | -4.331,20.241 | 0.199 | 0.054 | -14.454,14.562 | 0.994 |
| 4 | Index: Rule of law | -9.07 | -25.776,7.637 | 0.274 | 3.219 | -15.936,22.373 | 0.737 | -0.601 | -23.589,22.387 | 0.957 |
| 5 | Index: Regulatory quality | -5.839 | -20.262,8.585 | 0.412 | -4.793 | -21.631,12.045 | 0.570 | -5.853 | -31.350,19.644 | 0.639 |
| 6 | Index: Voice and accountability | -5.715 | -16.669,5.239 | 0.293 | 5.144 | -9.570,19.857 | 0.486 | -0.225 | -20.027,19.577 | 0.981 |
| 7 | Index: Logistics performance | -8.945 | -35.269,17.379 | 0.489 | 8.099 | -34.033,50.231 | 0.700 | 14.341 | -49.322,78.005 | 0.644 |
| 8 | Compliance with International Health Regulations | 0.122 | -0.546,0.790 | 0.710 | 0.289 | -0.297,0.875 | 0.327 | 0.042 | -0.658,0.741 | 0.903 |
| Health Information Systems | |  | | |  | | |  | | |
| 1 | Completeness of birth registration (%) | 0.229 | -0.585,1.043 | 0.564 | 0.207 | -0.275,0.690 | 0.391 | -0.216 | -0.646,0.213 | 0.308 |
| 2 | Malaria surveillance report completeness (%) | -87.273 | -261.114,86.568 | 0.310 | 74.673 | -14.212,163.557 | 0.098 | 33.286 | -30.690,97.262 | 0.292 |
| *Notes:*  *Interpretation of β: positive coefficient means the variable is positively associated with progress in malaria control (defined as reduction in malaria cases), and negative coefficient means the variable is negatively associated with progress in malaria control*  *Significance levels: * = significant at 0.05 level, **=significant at 0.01 level, *** = significant at 0.001 level* | | | | | | | | | | |
