# Supplementary material for: Health systems and global progress towards malaria elimination, 2000–2016
Source: Malar J. 2020 Apr 8;19:141. doi: 10.1186/s12936-020-03208-6 (PMC7140365; doi:10.1186/s12936-020-03208-6)
Supplement: Supplementary file 9 — Additional file 9. Country codes, health system scores, and percent reduction in malaria cases. [file 12936_2020_3208_MOESM9_ESM.docx]

**Additional file 9.** Percent reduction in Malaria Cases and Health System Scores, 2000-2016, according to the initial category of malaria case burden in 2000.

Health system scores are generated as percentile dimensions for 7 principal components (health service delivery, access to medicines, workforce, basic health center capacity, hospital capacity, governance and health information systems), so scores can range from 0 (weakest health system on all 7 components) to 70 (strongest health system on all 7 dimensions).

| # | Country Code | Country | % Reduction in Malaria Cases | **Overall HS Score** | Service Delivery | Access to Medicines | Health workforce | Health centre capacity | Hospital capacity | Governance | Health information systems |
| --- | --- | --- | --- | --- | --- | --- | --- | --- | --- | --- | --- |
| **High-Burden Countries (300+ cases per 1000 in 2000)** | | | | | | | | | | | |
| 1 | GNB | Guinea-Bissau | 78.8 | **44** | 3 | 8 | 7 | 10 | 10 | 3 | 3 |
| 2 | SLB | Solomon Islands | 77.9 | **41** | 7 | 5 | 6 | 8 | 7 | 5 | 3 |
| 3 | BDI | Burundi | 64.7 | **29** | 4 | 7 | 1 | 5 | 5 | 2 | 5 |
| 4 | GMB | The Gambia | 63.3 | **38** | 7 | 7 | 5 | 6 | 4 | 6 | 3 |
| 5 | TZA | Tanzania | 61.3 | **36** | 5 | 7 | 2 | 6 | 7 | 7 | 2 |
| 6 | UGA | Uganda | 60.4 | **31** | 3 | 9 | 3 | 5 | 3 | 6 | 2 |
| 7 | CIV | Côte d'Ivoire | 52.7 | **26** | 3 | 2 | 2 | 9 | 3 | 3 | 4 |
| 8 | ZMB | Zambia | 44.5 | **40** | 6 | 8 | 4 | 7 | 6 | 7 | 2 |
| 9 | COD | Democratic Republic of the Congo | 42.1 | **16** | 2 | 3 | 3 | 2 | 3 | 1 | 2 |
| 10 | LBR | Liberia | 39.0 | **21** | 2 | 7 | 1 | 4 | 3 | 3 | 1 |
| 11 | MWI | Malawi | 38.1 | **29** | 5 | 4 | 4 | 3 | 4 | 7 | 2 |
| 12 | CMR | Cameroon | 37.2 | **22** | 3 | 2 | 2 | 3 | 5 | 4 | 3 |
| 13 | COG | Congo | 36.8 | **41** | 5 | 10 | 3 | 8 | 8 | 2 | 5 |
| 14 | GAB | Gabon | 36.5 | **44** | 3 | 8 | 9 | 7 | 9 | 5 | 3 |
| 15 | MOZ | Mozambique | 36.2 | **18** | 3 | 2 | 1 | 1 | 1 | 6 | 4 |
| 16 | GHA | Ghana | 30.0 | **39** | 5 | 5 | 5 | 7 | 4 | 9 | 4 |
| 17 | CAF | Central African Republic | 25.2 | **14** | 1 | 1 | 2 | 4 | 4 | 1 | 1 |
| 18 | BFA | Burkina Faso | 24.9 | **32** | 3 | 4 | 1 | 9 | 2 | 7 | 6 |
| 19 | NGA | Nigeria | 21.4 | **23** | 1 | 3 | 6 | 8 | 1 | 3 | 1 |
| 20 | SLE | Sierra Leone | 21.1 | **22** | 4 | 7 | 1 | 1 | 1 | 3 | 5 |
| 21 | GNQ | Equatorial Guinea | 20.7 | **24** | 1 | 5 | 4 | 1 | 8 | 1 | 4 |
| 22 | TGO | Togo | 13.9 | **26** | 3 | 2 | 1 | 10 | 3 | 3 | 4 |
| 23 | GIN | Guinea | 6.5 | **16** | 1 | 1 | 2 | 5 | 1 | 3 | 3 |
| 24 | MLI | Mali | -3.5 | **23** | 2 | 2 | 1 | 7 | 1 | 6 | 4 |
| 25 | NER | Niger | -18.0 | **21** | 1 | 1 | 2 | 7 | 1 | 6 | 3 |
| **Middle-Burden Countries (1-300 cases per 1000 in 2000)** | | | | | | | | | | | |
| 1 | LKA | Sri Lanka | 100.0 | **53** | 10 | 2 | 7 | 10 | 9 | 8 | 7 |
| 2 | PRY | Paraguay | 100.0 | **46** | 9 | 7 | 8 | 4 | 6 | 5 | 7 |
| 3 | TJK | Tajikistan | 100.0 | **55** | 6 | 9 | 10 | 9 | 10 | 3 | 8 |
| 4 | TLS | Timor-Leste | 99.9 | **42** | 2 | 7 | 3 | 10 | 10 | 5 | 5 |
| 5 | BLZ | Belize | 99.8 | **54** | 9 | 6 | 7 | 9 | 6 | 8 | 9 |
| 6 | BTN | Bhutan | 99.8 | **45** | 8 | 3 | 5 | 6 | 7 | 9 | 7 |
| 7 | SUR | Suriname | 99.4 | **59** | 7 | 8 | 9 | 8 | 9 | 9 | 9 |
| 8 | ECU | Ecuador | 99.1 | **38** | 6 | 4 | 8 | 2 | 5 | 5 | 8 |
| 9 | VNM | Vietnam | 98.0 | **52** | 7 | 8 | 5 | 8 | 8 | 7 | 9 |
| 10 | COM | Comoros | 97.9 | **32** | 4 | 4 | 3 | 4 | 7 | 3 | 7 |
| 11 | PRK | North Korea | 97.3 | **56** | 8 | 10 | 10 | 7 | 10 | 1 | 10 |
| 12 | BWA | Botswana | 97.0 | **52** | 8 | 5 | 7 | 10 | 7 | 10 | 5 |
| 13 | GTM | Guatemala | 95.6 | **38** | 7 | 6 | 5 | 2 | 2 | 6 | 10 |
| 14 | STP | Sao Tome and Principe | 95.1 | **54** | 8 | 10 | 8 | 5 | 9 | 7 | 7 |
| 15 | KHM | Cambodia | 94.2 | **35** | 4 | 10 | 3 | 6 | 2 | 4 | 6 |
| 16 | NPL | Nepal | 94.1 | **29** | 4 | 3 | 7 | 2 | 6 | 4 | 3 |
| 17 | HND | Honduras | 92.2 | **43** | 7 | 9 | 6 | 4 | 3 | 5 | 9 |
| 18 | MMR | Myanmar | 91.0 | **26** | 5 | 6 | 5 | 3 | 2 | 1 | 4 |
| 19 | BOL | Bolivia | 90.8 | **41** | 6 | 5 | 5 | 8 | 5 | 6 | 6 |
| 20 | PHL | Philippines | 89.9 | **45** | 5 | 4 | 9 | 9 | 4 | 8 | 6 |
| 21 | VUT | Vanuatu | 89.8 | **47** | 4 | 7 | 7 | 10 | 9 | 9 | 1 |
| 22 | BRA | Brazil | 89.3 | **64** | 10 | 10 | 10 | 7 | 9 | 10 | 8 |
| 23 | LAO | Laos | 88.8 | **33** | 1 | 5 | 4 | 9 | 5 | 3 | 6 |
| 24 | THA | Thailand | 86.6 | **58** | 8 | 9 | 5 | 9 | 8 | 9 | 10 |
| 25 | SEN | Senegal | 80.0 | **25** | 4 | 3 | 1 | 3 | 1 | 8 | 5 |
| 26 | NIC | Nicaragua | 79.0 | **44** | 8 | 6 | 7 | 5 | 4 | 6 | 8 |
| 27 | AFG | Afghanistan | 78.1 | **19** | 1 | 8 | 4 | 2 | 1 | 1 | 2 |
| 28 | ETH | Ethiopia | 71.4 | **18** | 1 | 1 | 2 | 4 | 5 | 4 | 1 |
| 29 | NAM | Namibia | 69.5 | **52** | 6 | 6 | 9 | 5 | 9 | 10 | 7 |
| 30 | KEN | Kenya | 65.5 | **34** | 4 | 8 | 2 | 6 | 6 | 6 | 2 |
| 31 | HTI | Haiti | 61.9 | **20** | 2 | 1 | 6 | 3 | 4 | 2 | 2 |
| 32 | ZWE | Zimbabwe | 58.6 | **32** | 6 | 2 | 5 | 7 | 7 | 1 | 4 |
| 33 | IND | India | 55.3 | **44** | 3 | 10 | 7 | 5 | 4 | 9 | 6 |
| 34 | SDN | Sudan | 53.4 | **24** | 4 | 3 | 5 | 5 | 4 | 1 | 2 |
| 35 | COL | Colombia | 51.9 | **48** | 9 | 8 | 6 | 1 | 6 | 8 | 10 |
| 36 | DJI | Djibouti | 50.5 | **25** | 4 | 1 | 2 | 8 | 2 | 4 | 4 |
| 37 | AGO | Angola | 49.9 | **28** | 2 | 5 | 6 | 6 | 6 | 2 | 1 |
| 38 | PAK | Pakistan | 49.7 | **25** | 2 | 9 | 4 | 2 | 3 | 4 | 1 |
| 39 | SSD | South Sudan | 42.7 | **13** | 1 | 1 | 1 | 7 | 1 | 1 | 1 |
| 40 | PNG | Papua New Guinea | 37.1 | **34** | 3 | 5 | 4 | 9 | 4 | 5 | 4 |
| 41 | IDN | Indonesia | 36.8 | **34** | 5 | 9 | 3 | 5 | 2 | 7 | 3 |
| 42 | SOM | Somalia | 36.6 | **14** | 1 | 1 | 3 | 1 | 6 | 1 | 1 |
| 43 | GUY | Guyana | 36.2 | **49** | 9 | 4 | 6 | 9 | 9 | 7 | 5 |
| 44 | TCD | Chad | 26.5 | **10** | 1 | 1 | 1 | 2 | 2 | 2 | 1 |
| 45 | PER | Peru | 24.4 | **47** | 9 | 6 | 6 | 5 | 5 | 8 | 8 |
| 46 | MRT | Mauritania | 14.4 | **22** | 2 | 3 | 3 | 6 | 2 | 4 | 2 |
| 47 | MDG | Madagascar | 5.5 | **22** | 2 | 4 | 1 | 4 | 1 | 5 | 5 |
| 48 | YEM | Yemen | 0.6 | **23** | 2 | 4 | 3 | 6 | 5 | 2 | 1 |
| 49 | BEN | Benin | 0.5 | **30** | 5 | 1 | 2 | 7 | 2 | 7 | 6 |
| 50 | ERI | Eritrea | -4.2 | **24** | 5 | 3 | 4 | 2 | 3 | 2 | 5 |
| 51 | RWA | Rwanda | -94.6 | **39** | 6 | 3 | 7 | 4 | 8 | 6 | 5 |
| 52 | VEN | Venezuela | -326.1 | **35** | 7 | 6 | 8 | 1 | 3 | 3 | 7 |
| **Low-Burden Countries (<1 case per 1000 in 2000)** | | | | | | | | | | | |
| 1 | TKM | Turkmenistan | 100.0 | **47** | 10 | 7 | 10 | 1 | 10 | 2 | 7 |
| 2 | ARM | Armenia | 100.0 | **48** | 9 | 2 | 10 | 3 | 10 | 8 | 6 |
| 3 | AZE | Azerbaijan | 100.0 | **54** | 6 | 5 | 10 | 9 | 10 | 5 | 9 |
| 4 | UZB | Uzbekistan | 100.0 | **49** | 10 | 8 | 10 | 1 | 10 | 2 | 8 |
| 5 | OMN | Oman | 100.0 | **58** | 10 | 9 | 9 | 3 | 7 | 10 | 10 |
| 6 | SYR | Syria | 100.0 | **33** | 6 | 3 | 8 | 4 | 5 | 2 | 5 |
| 7 | TUR | Turkey | 100.0 | **52** | 8 | 7 | 8 | 3 | 9 | 9 | 8 |
| 8 | ARG | Argentina | 100.0 | **64** | 9 | 9 | 9 | 10 | 9 | 8 | 10 |
| 9 | GEO | Georgia | 100.0 | **52** | 8 | 5 | 10 | 3 | 9 | 9 | 8 |
| 10 | IRQ | Iraq | 100.0 | **32** | 5 | 4 | 5 | 5 | 5 | 1 | 7 |
| 11 | DZA | Algeria | 100.0 | **37** | 8 | 4 | 7 | 6 | 2 | 4 | 6 |
| 12 | KGZ | Kyrgyzstan | 100.0 | **49** | 9 | 6 | 10 | 1 | 10 | 4 | 9 |
| 13 | MAR | Morocco | 100.0 | **39** | 7 | 3 | 4 | 8 | 3 | 8 | 6 |
| 14 | CHN | China | 99.97 | **51** | 8 | 1 | 9 | 10 | 8 | 8 | 7 |
| 15 | CRI | Costa Rica | 99.8 | **50** | 9 | 8 | 6 | 2 | 5 | 10 | 10 |
| 16 | IRN | Iran | 99.7 | **50** | 10 | 6 | 7 | 6 | 7 | 4 | 10 |
| 17 | MYS | Malaysia | 98.4 | **62** | 10 | 10 | 8 | 9 | 6 | 10 | 9 |
| 18 | SLV | El Salvador | 98.4 | **45** | 9 | 6 | 6 | 3 | 3 | 9 | 9 |
| 19 | SAU | Saudi Arabia | 97.4 | **55** | 10 | 9 | 9 | 1 | 8 | 9 | 9 |
| 20 | BGD | Bangladesh | 94.4 | **19** | 3 | 2 | 3 | 2 | 1 | 5 | 3 |
| 21 | MEX | Mexico | 94.1 | **62** | 10 | 7 | 9 | 10 | 8 | 9 | 9 |
| 22 | KOR | South Korea | 86.8 | **69** | 10 | 10 | 9 | 10 | 10 | 10 | 10 |
| 23 | ZAF | South Africa | 80.7 | **52** | 5 | 10 | 9 | 3 | 8 | 10 | 7 |
| 24 | CPV | Cape Verde | 73.1 | **48** | 6 | 5 | 4 | 8 | 7 | 10 | 8 |
| 25 | SWZ | Eswatini | 64.9 | **38** | 7 | 2 | 10 | 4 | 7 | 5 | 3 |
| 26 | DOM | Dominican Republic | 52.5 | **53** | 7 | 9 | 8 | 7 | 7 | 7 | 8 |
| 27 | PAN | Panama | 44.3 | **61** | 9 | 9 | 8 | 8 | 8 | 10 | 9 |
| 28 | EGY | Egypt | 0.0* | **49** | 7 | 10 | 8 | 1 | 6 | 7 | 10 |
| ***Notes:***  *Egypt had 0 cases in 2000 and 2016 (the only cases during the entire period were in 2014, when there were 22 cases) | | | | | | | | | | | |
