# Supplementary material for: Health systems and global progress towards malaria elimination, 2000–2016
Source: Malar J. 2020 Apr 8;19:141. doi: 10.1186/s12936-020-03208-6 (PMC7140365; doi:10.1186/s12936-020-03208-6)
Supplement: Supplementary file 10 — Additional file 10. Full model selection results with p-values. [file 12936_2020_3208_MOESM10_ESM.docx]

**Additional file 10.** Full model selection results with p-values.

**Table 3: Model Results (coefficients and R-squared) for malaria case reductions and health system components, 2000-2016.**

| Health System Component | | Percent reduction in cases  per capita, 2000-2016 | | | | | | Probability of reaching  0 cases by 2016 ^†^ | | | | | |  |  |
| --- | --- | --- | --- | --- | --- | --- | --- | --- | --- | --- | --- | --- | --- | --- | --- |
| Model Selection Method | | All  (ß) | *p* | Backward Stepwise (ß) | *p* | Grid Search  (ß) | *p* | All  (ß) | *p* | Backward Stepwise  (ß) | *p* | Grid Search  (ß) | *p* | |  |
| 1 | Health service delivery, routine services | 4.206 | *0.223* | 5.135 | *0.073* | 4.938 | *0.085* | -0.047 | *0.675* | Not selected. | *--* | Not selected. | *--* | |  |
| 2 | Access to medicines | 4.345 | *0.294* | Not selected. | *--* | Not selected. | *--* | -0.131 | *0.144* | Not selected. | *--* | Not selected. | *--* | |  |
| 3 | Health system workforce | 0.606 | *0.762* | Not selected. | *--* | Not selected. | *--* | 0.015 | *0.844* | Not selected. | *--* | Not selected. | *--* | |  |
| 4 | Health system capacity: health centers | 6.657 | *0.198* | Not selected. | *--* | 6.587 | *0.269* | -0.102* | *0.027* | -0.099* | *0.014* | -0.099* | *0.014* | |  |
| 5 | Health system capacity: hospitals | 6.091 | *0.125* | 9.912* | *0.041* | 6.917* | *0.014* | 0.179 | *0.230* | 0.204** | *0.007* | 0.204** | *0.007* | |  |
| 6 | Governance | 1.042 | *0.781* | Not selected. | *--* | Not selected. | *--* | -0.054 | *0.375* | -0.075 | *0.111* | -0.075 | *0.111* | |  |
| 7 | Health information systems | -0.467 | *0.914* | Not selected. | *--* | Not selected. | *--* | 0.271 | *0.291* | Not selected. | *--* | Not selected. | *--* | |  |
| R^2^ for model: | | .204 | | .174 | | .197 | | .454 | | .413 | | .413 | | |  |
| *Notes:* All regressions adjust for HDI in 2000. Backward stepwise models exclude variables with p>0.2 and include variables with p-values < 0.10. Grid search results reflect the model with lowest root mean squared error, among all models including up to 7 components.  ^†^Analysis of countries which reached 0 cases is conducted among countries in with low initial burden (<1 case per 1000 in 2000) only.  *: Variable is significant at p<.05; **: Variable is significant at p<.01 | | | | | | | | | | | | | | |  |

**Table 4: Backward stepwise model results (coefficients and R-squared) for malaria case reductions and health system components, 2000-2016, stratified by burden category.**

| Health System Component | | Percent reduction in cases per capita, 2000-2016  (ß) | | | | | |  |
| --- | --- | --- | --- | --- | --- | --- | --- | --- |
| Model Selection Method | | Low  (ß) | *p* | Middle  (ß) | *p* | High  (ß) | *p* |  |
| 1 | Health service delivery, routine services | Not selected. | *--* | Not selected. | *--* | 17.165** | *0.000* |  |
| 2 | Access to medicines | -12.355 | *0.142* | 12.079 | *0.065* | Not selected. | *--* |  |
| 3 | Health system workforce | Not selected. | *--* | Not selected. | *--* | Not selected. | *--* |  |
| 4 | Health system capacity: health centers | -1.973 | *0.052* | 20.030 | *0.123* | Not selected. | *--* |  |
| 5 | Health system capacity: hospitals | 5.550 | *0.120* | Not selected. | *--* | 6.565** | *0.000* |  |
| 6 | Governance | Not selected. | *--* | Not selected. | *--* | -4.480 | *0.068* |  |
| 7 | Health information systems | Not selected. | *--* | Not selected. | *--* | -9.323** | *0.003* |  |
| R^2^ for model: | | .258 | | .159 | | .705 | |  |

***Notes:*** All regressions adjust for initial HDI in 2000. Backward stepwise models exclude variables with p>0.2 and include variables with p-values <.1.

^†^Initial Burden Category is defined as: Low = <1 case per 1000 in 2000, Middle = 1-300 cases per 1000 in 2000, High = 300+ cases per 1000 in 2000 ;*: Variable is significant at p<.05; **: Variable is significant at p<.01
